# Cerebrolysin, hemorrhagic transformation, and anticoagulation timing after reperfusion therapy in stroke: *post hoc* secondary analysis of the CEREHETIS trial

**DOI:** 10.3389/fphar.2025.1725255

**Published:** 2026-01-07

**Authors:** Mikhail N. Kalinin, Dina R. Khasanova

**Affiliations:** 1 Department of Neurology, Kazan State Medical University, Kazan, Russia; 2 Department of Neurology, Interregional Clinical Diagnostic Center, Kazan, Russia

**Keywords:** anticoagulation timing, Cerebrolysin, hemorrhagic transformation, intravenous thrombolysis, stroke, survival analaysis, compounding effect, nonlinear hazard acceleration

## Abstract

**Background:**

The optimal timing of anticoagulation resumption after acute ischemic stroke remains uncertain, particularly in patients at elevated risk of hemorrhagic transformation (HT). Although Cerebrolysin reduces HT incidence, its influence on dynamic HT risk and the safe anticoagulation window remains unclear.

**Methods:**

This *post hoc* secondary survival analysis of the CEREHETIS trial (ISRCTN87656744) included 238 patients with intravenous thrombolysis (IVT)–treated middle cerebral artery (MCA) infarction. Patients were categorized into low (HTI 0) and high (HTI 1–4) HT-risk groups. Fourteen-day HT hazard trajectories were modeled using the Gompertz distribution. Nonlinear hazard acceleration (NLHA) and the compounding effect—reflecting self-amplifying instantaneous risk—were used to identify the inception point at which hazard stabilization may permit anticoagulation. A conservative NLHA threshold (0.23%–0.44%/day) defined this risk-equilibrium.

**Results:**

In high-risk patients, Cerebrolysin significantly reduced symptomatic HT (HR 0.245; 95% CI 0.072–0.837; *p* = 0.020) and any HT (HR 0.543; 95% CI 0.297–0.991; *p* = 0.032). In controls, the compounding effect peaked on day 1 and persisted into day 2, whereas Cerebrolysin attenuated this amplification and shortened the hazardous period. Inception points occurred on days 1–3 with Cerebrolysin vs. days 3–5 in controls. In low-risk patients, both groups reached stable hazard by day 2.

**Conclusion:**

In IVT-treated MCA stroke patients with HTI 1–4, Cerebrolysin may reduce HT hazard and advance the risk-equilibrium point by approximately 1–2 days. These findings are preliminary and hypothesis-generating, suggesting that Cerebrolysin may allow earlier—but individualized—anticoagulation resumption in selected high-risk patients, pending prospective validation.

## Graphical Abstract

**Figure:**
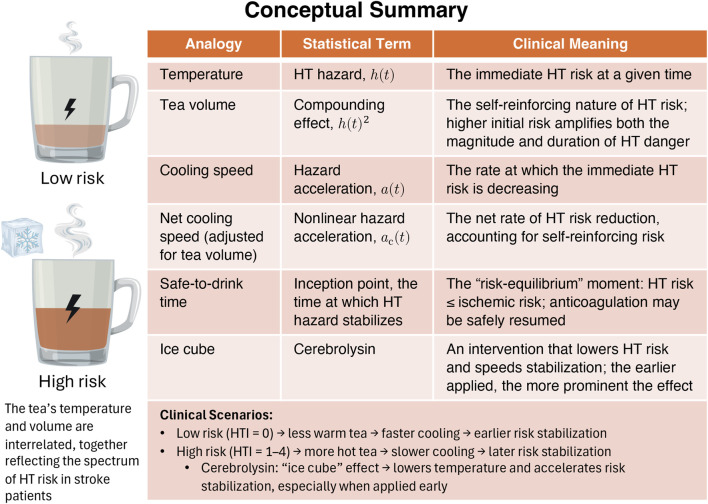

## Introduction

Recent advances in reperfusion therapy, including third-generation thrombolytic agents and endovascular thrombectomy, have profoundly transformed stroke management (Tsivgoulis et al., 2023; Raha et al., 2023). Despite these developments, optimizing the efficacy and safety of intravenous thrombolysis (IVT) with alteplase—the current gold standard for acute ischemic stroke—remains a major clinical priority (Otsu et al., 2020). Combining multimodal cytoprotective agents such as Cerebrolysin with IVT has emerged as a promising approach to enhance penumbral tissue survival, mitigate reperfusion injury, and reduce the risk of hemorrhagic transformation (HT) (Lang et al., 2013; Poljakovic et al., 2021; Khasanova and Kalinin, 2023a; Khasanova and Kalinin, 2023b; Kalinin and Khasanova, 2024a; Kalinin and Khasanova, 2024b).

Cerebrolysin is a purified porcine brain-derived preparation containing low-molecular-weight neuropeptides and free amino acids that act similarly to endogenous neurotrophic factors, supporting protection and repair of the neurovascular units (Masliah et al., 2012; Zhang et al., 2010; Teng et al., 2021). Clinical trials and meta-analyses have demonstrated that Cerebrolysin promotes early neurological recovery, reduces post-stroke deficits, and exhibits a favorable safety and tolerability profile (Lang et al., 2013; Guekht et al., 2017; Bornstein et al., 2018). Our original CEREHETIS study (Khasanova and Kalinin, 2023a; Khasanova and Kalinin, 2023b) and subsequent analyses (Kalinin and Khasanova, 2024a; Kalinin and Khasanova, 2024b) confirmed that the combination of Cerebrolysin with IVT is safe, decreases the incidence of HT, and improves functional outcomes in patients with moderate to high HT risk identified at admission.

However, the temporal characteristics of Cerebrolysin’s anti-HT effects and their implications for the timing of anticoagulation resumption in patients with atrial fibrillation (AF) remain unclear. Determining the optimal timing to restart anticoagulation after ischemic stroke is particularly challenging despite extensive research (Wilson et al., 2019; Sharobeam et al., 2024; Fischer et al., 2023). Clinicians face a critical balance: initiating therapy too early increases the risk of intracranial hemorrhage, whereas delaying it may elevate the likelihood of recurrent ischemic events.

To address these gaps, we conducted a *post hoc* secondary survival analysis of the CEREHETIS trial to characterize the temporal dynamics of HT hazard in patients with varying predicted HT risk. Based on these findings, we developed a hazard-based analytical framework to determine the optimal timing for anticoagulation resumption after ischemic stroke. By quantifying instantaneous and evolving hemorrhagic risks, this approach enables clinicians to balance competing ischemic and hemorrhagic hazards in real time, effectively translating complex survival dynamics into actionable therapeutic windows.

## Methods

CEREHETIS was a prospective, randomized, open-label, active control, multicenter, parallel-group, phase IIIb pilot study (trial registration number: ISRCTN87656744). The protocol, patient inclusion and exclusion criteria, and the original results have been published previously (Khasanova and Kalinin, 2023a; Khasanova and Kalinin, 2023b).

Enrollment took place across eight centers in Russia. Each eligible patient was randomly assigned to either the Cerebrolysin group or the control group. Both arms received a standard intravenous dose of 0.9 mg/kg alteplase (Actilyse®, Boehringer Ingelheim GmbH, Germany) within 4.5 h of symptom onset. Of this dose, 10% was administered as a bolus, while the remainder was infused over 60 min, with the maximum dosage not exceeding 90 mg. Patients in the intervention group additionally received 30 mL of Cerebrolysin® (EVER Pharma GmbH, Austria) mixed in 100 mL of normal saline, infused intravenously via a separate line over 20 min. This treatment was initiated simultaneously with IVT and continued once daily for 14 consecutive days.

In patients with non-valvular AF, secondary stroke prevention with a non-vitamin K antagonist oral anticoagulant (NOAC) was initiated between days 3 and 6, following the 1-3-6-12-day rule (Steffel et al., 2021), irrespective of treatment group. Subsequent references to the “timing of restarting anticoagulation” specifically denote the resumption window for NOAC therapy in these patients.

Failure events were defined as any HT and symptomatic HT. These events were verified by scheduled follow-up computed tomography scans at 24 h, on days 7 and 14, or at any time upon a clinician’s request. The time scale was measured in days from admission, with HT occurring within 24 h after IVT credited to day 1. Symptomatic HT was defined according to the ECASS III trial as any extravascular blood in the brain or within the cranium associated with clinical deterioration, indicated by an increase of four points or more on the National Institutes of Health Stroke Scale (NIHSS), or leading to death and identified as the predominant cause of neurological deterioration (Hacke et al., 2008).

The analysis period was limited to 14 consecutive days, with right-censoring applied to patients who did not experience failure. Drop-out participants were censored on the day of their exit. Subjects with any HT and symptomatic HT were considered to have failed on the day HT was confirmed.

### Study measurements

The present study utilized the same cohort as our previous research (Kalinin and Khasanova, 2024a; Kalinin and Khasanova, 2024b). Patients with middle cerebral artery (MCA) infarction were selected from the intention-to-treat population of the CEREHETIS trial. MCA stroke patients were chosen to ensure anatomical homogeneity and to minimize variability in treatment effects related to differences between anterior and posterior circulation. Previous studies (Merwick and Werring, 2014; Sung et al., 2013) have shown that several HT risk assessment tools perform less reliably in posterior circulation strokes, further supporting the focus on MCA infarctions.

The Hemorrhagic Transformation Index (HTI) score was applied to estimate the risk of HT upon admission, consistent with our earlier studies (Kalinin and Khasanova, 2024a; Kalinin and Khasanova, 2024b), which identified the HTI as the most reliable predictor of HT risk within this cohort. The HTI was defined according to the original description (Kalinin et al., 2017) as follows: ASPECTS: 10–7 = 0; 6–5 = 1; 4–3 = 2; 2–0 = 3; NIHSS: 0–11 = 0; 12–17 = 1; 18–23 = 2; >23 = 3; hyperdense MCA sign: yes = 1; AF on ECG upon admission: yes = 1. The total score ranged from 0 to 8, with each one-point increase indicating a higher probability of HT. Although the HTI was originally developed and externally validated (de Andrade et al., 2022) to predict any HT in MCA stroke patients irrespective of IVT status, it has also demonstrated predictive value for IVT-related symptomatic HT (Kalinin and Khasanova, 2024a; Kalinin and Khasanova, 2024b).

For each included patient, the HTI score was retrospectively calculated from admission data and used to classify patients into two subgroups: low (HTI 0) and high (HTI 1–4) HT risk, as patients scoring 1–4 were shown to derive the greatest benefit from Cerebrolysin treatment (Kalinin and Khasanova, 2024a; Kalinin and Khasanova, 2024b). Patients with very high HT risk (HTI >4) were not represented in the cohort because they were ineligible for IVT and therefore were not enrolled in the parent trial.

### Statistical analysis

Data analysis was conducted using StataNow/SE v.19.5 (StataCorp LLC, United States). Descriptive statistics included the median (M) with interquartile range (IQR) for non-normally distributed continuous variables and percentages for categorical variables. Group comparisons of baseline characteristics were performed using the Mann–Whitney *U* test for continuous variables and Pearson’s χ^2^ test for categorical variables.

Survival analysis was applied to model the time to failure events. Key definitions are as follows:

- Survival function $S\left(t\right)$ : The probability that a patient remains free from HT up to time t .
- Restricted mean survival time (RMST): The mean survival time up to a prespecified time horizon $t^{*}$ . In clinical studies, the treatment effect is quantified as the difference in RMST between treatment groups at $t^{*}$ . RMST is estimated as the area under the survival curve from 0 to $t^{*}$ (Syriopoulou et al., 2022; Royston, 2015).
- Hazard function $h\left(t\right)$ : The instantaneous rate of HT at time t , conditional on survival up to that time, defined as (Hess and Levin, 2014):

$$h\left(t\right)=-\frac{S^{\prime }\left(t\right)}{S\left(t\right)}$$

- Compounding effect: Describes the self-reinforcing nature of instantaneous risk over time, reflecting the nonlinear escalation of hazard. It is modeled as a quadratic function of the hazard $h{\left(t\right)}^{2}$ , capturing the feedback mechanism through which elevated risk levels amplify their own impact (Yashin et al., 2012; Bhatti et al., 2018).
- Hazard acceleration $a\left(t\right)$ : The rate of change in the hazard function over time, reflecting how quickly the instantaneous risk evolves. Because the probability of HT typically decreases over time (Kalinin et al., 2019), the derivative of the hazard function becomes negative; therefore, a minus sign is applied to express acceleration as a positive value (Kalinin and Khasanova, 2025):

$$a\left(t\right)=-h^{\prime }\left(t\right)$$

- Nonlinear hazard acceleration (NLHA) $a_{c}\left(t\right)$ : Extends hazard acceleration $a\left(t\right)$ by incorporating the compounding effect, which captures self-reinforcing risk dynamics and nonlinear amplification (Kalinin and Khasanova, 2025):

$$a_{c}\left(t\right)=a\left(t\right)+h{\left(t\right)}^{2}$$

- Inception point: The time point at which the risk of HT is no longer imminent and the competing risk of recurrent ischemic events predominates, allowing safe resumption of anticoagulation. Parametrically, it corresponds to the time t when hazard acceleration approaches zero and the hazard function stabilizes. The preceding interval is referred to as the hazardous period (Kalinin and Khasanova, 2025).
- Standardized curve: The mean of the predicted curves across all observations in the dataset (Syriopoulou et al., 2022).
- Difference in the curves: The contrast between two hypothetical scenarios—one in which all patients receive Cerebrolysin and one in which none do (Syriopoulou et al., 2022). The resulting difference in survival represents the absolute risk reduction (ARR).

The survival function was adjusted for treatment assignment (Cerebrolysin vs. control) and HT risk subgroups (low vs. high), followed by nonparametric, semiparametric, and parametric analyses. Kaplan–Meier survival curves were generated and compared using the log-rank test. If the proportional hazards (PH) assumption was violated, a combined test with 5,000 permutations was additionally applied to confirm the results (Royston, 2017). A Cox PH model was then fitted, with tied failures handled using the Efron method for its superior performance (Hertz-Picciotto and Rockhill, 1997). The PH assumption was assessed using graphical diagnostics and the Schoenfeld residuals test. When the assumption was violated, a time-varying Cox model was applied, and time-dependent (TD) hazard ratios (HR) with 95% confidence intervals (CI) were computed. Several parametric models were subsequently fitted, with model selection guided by the Akaike and Bayesian information criteria. Ancillary parameters were included as necessary to refine the hazard shape, and standardized curves with corresponding 95% CIs were generated (Syriopoulou et al., 2022). Model adequacy was evaluated using post-estimation goodness-of-fit tests.

The effect of Cerebrolysin treatment on HT was evaluated parametrically using HR, population attributable fraction, ARR, number needed to treat (NNT), and RMST difference (Syriopoulou et al., 2022; Royston, 2015; Newson, 2013). NNT was derived from the formula (Yang and Yin, 2019):

$$\text{NNT}\left(t\right)=\frac{1}{\text{ARR}}$$

HR *p*-values were adjusted for multiple hypothesis testing using the Romano–Wolf method with 1,000 bootstrap replications (Clarke et al., 2020).

The Hausman specification test was applied to compare the coefficients of the parametric models for any HT and symptomatic HT in order to assess whether the estimates were statistically equivalent. When no significant difference was detected (*p* ≥ 0.05), the model for symptomatic HT was used to guide anticoagulation timing, as this outcome is clinically more relevant. Conversely, if the coefficients significantly differed (*p* < 0.05), the consistent model was preferred to minimize potential bias in timing estimation. The Hausman test was also employed to compare coefficients of semiparametric and parametric models as a part of the post-estimation goodness-of-fit assessment.

To identify the boundary at which HT risk becomes time-invariant, the NLHA threshold was derived from the most vulnerable subgroup—control patients with high HT risk—using the 95%-CI lower-bound NLHA trajectory. This approach provided a conservative, safety-oriented estimate that minimized the risk of overlooking clinically relevant HT. Upper 95%-CI bounds were not used because they represent maximal uncertainty and would artificially delay the estimated time to safe anticoagulation, increasing ischemic risk. Using the lower-bound NLHA curve therefore identified the earliest time at which HT risk becomes clinically negligible, balancing hemorrhagic safety with timely anticoagulation.

**FIGURE 1:**
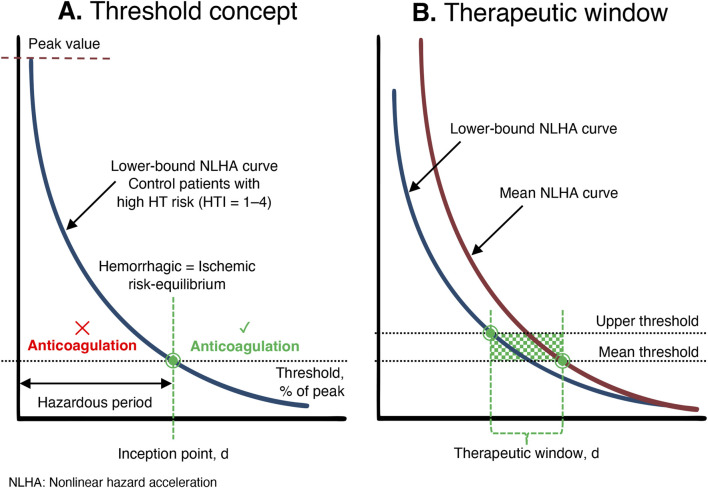
Analytical framework for assessing hazard dynamics of HT to determine the optimal timing for anticoagulation resumption. Panel **(A)** Threshold concept. Panel **(B)** Therapeutic window for anticoagulation resumption.

The hazard function reflects instantaneous HT risk but does not describe how that risk evolves. Hazard acceleration quantifies the rate at which the hazard changes over time; when it approaches zero, the trajectory has stabilized. NLHA extends this concept by incorporating self-amplifying effects, capturing both the magnitude of risk and the dynamics governing its evolution. Because NLHA identifies the time at which risk propagation effectively ceases, it provides the most clinically meaningful marker of when anticoagulation can be safely resumed. Together, these metrics translate complex survival modeling into a clinically intuitive framework: an initial phase of unstable HT risk (the hazardous period) followed by stabilization (the inception point), enabling estimation of the earliest safe anticoagulation window for each HT-risk subgroup (Figure 1).

Because the risk-equilibrium point cannot be known *a priori*, several candidate thresholds were evaluated. Thresholds were defined as fixed proportions of the peak 95%-CI lower-bound NLHA value (2%, 5%, 10%, and 13%), corresponding to hazard levels within the reported incidence of early ischemic recurrence in patients with acute stroke and AF (0.1%–1.3%/day) (Paciaroni et al., 2016). These proportions sampled the low, mid, and upper segments of the clinically relevant range without overspecifying the grid. For each threshold, two inception points were calculated: (i) the time at which the 95%-CI lower-bound NLHA curve crossed the threshold and (ii) the time at which the mean NLHA curve crossed it. This sensitivity analysis quantified how threshold selection influences the estimated duration of the hazardous period.

To select an analytically justified cut-off, each threshold θ was evaluated using three quantitative criteria. First, the central inception estimate $T_{c}\left(\theta \right)$ summarized the midpoint between lower-bound and mean stabilization times ( $T_{lb}\left(\theta \right)$ and $T_{mean}\left(\theta \right)$ , respectively):

$$T_{c}\left(\theta \right)=\frac{T_{lb}\left(\theta \right)+T_{mean}\left(\theta \right)}{2}$$

Second, internal uncertainty $U\left(\theta \right)$ quantified the spread between these two estimates:

$$U\left(\theta \right)=T_{mean}\left(\theta \right)-T_{lb}\left(\theta \right)$$

Third, global representativeness $D\left(\theta \right)$ measured how closely each threshold aligned with the overall pattern of inception points across all cut-offs:

$$D\left(\theta \right)=\left|T_{c}\left(\theta \right)-{\bar{T}}_{c}\right|$$

where ${\bar{T}}_{c}$ is the mean of all central inception estimates. The optimal threshold was defined as the value minimizing $D\left(\theta \right)$ .

After identifying the optimal point-estimated threshold, its uncertainty was quantified using the delta-method. Because the threshold was a fixed proportion of the NLHA peak, it was treated as a direct transformation of that peak. The variance of the transformed quantity was derived from the mean and 95%-CI bounds of the NLHA peak, yielding both a point estimate and corresponding 95% CIs for the threshold.

For determining inception points, only the upper-bound and mean threshold values were used. Applying the lower-bound threshold would delay the crossing of the NLHA trajectories and artificially prolong the hazardous period, producing an unduly conservative estimate. In contrast, the upper-bound threshold identifies the earliest clinically defensible stabilization point, while the mean threshold provides a central reference. Because HT risk decreases monotonically (Kalinin and Khasanova, 2025), each subgroup’s threshold is crossed exactly once, allowing unambiguous identification of inception times. The therapeutic window for anticoagulation resumption was defined as the interval between the earliest estimate (95%-CI lower-bound NLHA curve crossing the upper threshold) and the typical estimate (mean NLHA curve crossing the mean threshold) (Figure 1). All Stata code and raw data used in this study are provided in the Supplementary Material.

## Results

**FIGURE 2:**
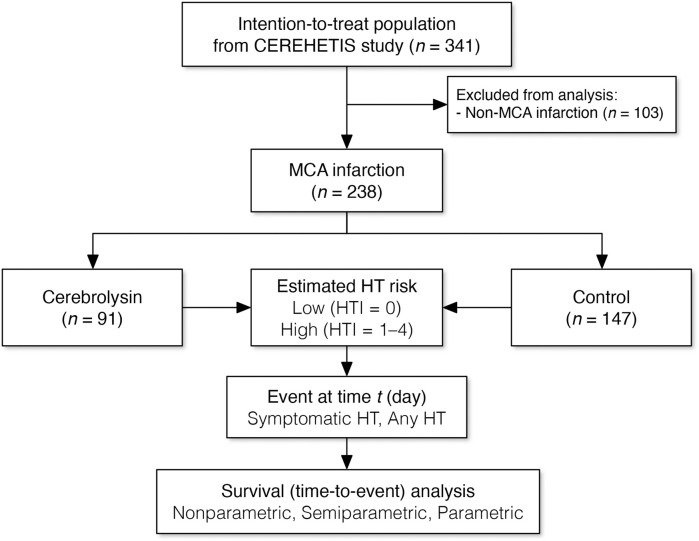
Study flow chart.

Although the intention-to-treat population of the CEREHETIS trial included 341 participants, only 238 had MCA infarction and were therefore analyzed (Figure 2). Nearly one-third of these patients had AF, but no recurrent strokes occurred during the follow-up period. More than half of the analyzed patients belonged to the high-HT-risk subgroup (HTI 1–4). At baseline, patients in the Cerebrolysin group were slightly younger and had fewer cases of previous stroke (Table 1). However, this covariate imbalance did not influence event rates, as confirmed by both semiparametric and parametric adjusted analyses.

**TABLE 1:** Baseline characteristics at admission (*n* = 238).

Characteristics	Cerebrolysin, *n* = 91	Control, *n* = 147	*p*-value
Age, yr (M, IQR)	64 (56–72)	69 (61–79)	0.006
Sex, male, *n* (%)	53 (58.2)	78 (53.1)	0.435
NIHSS (M, IQR)	11 (7–14)	11 (7–15)	0.687
ASPECTS (M, IQR)	10 (9–10)	10 (9–10)	0.668
Hyperdense MCA sign, *n* (%)	6 (6.6)	6 (4.1)	0.389
Atrial fibrillation, *n* (%)			
History	27 (29.7)	33 (22.5)	0.212
ECG (on admission)	25 (27.5)	38 (25.9)	0.783
Status (combined)	31 (34.1)	43 (29.3)	0.436
Diabetes mellitus, *n* (%)	17 (18.7)	22 (15.0)	0.452
Hypertension, *n* (%)	72 (79.1)	129 (87.8)	0.074
Previous stroke, *n* (%)	13 (14.3)	38 (25.9)	0.035
Previous use of antiplatelet agents, *n* (%)	24 (26.4)	39 (26.5)	0.979
Systolic blood pressure, mm Hg (M, IQR)	150 (138–165)	150 (140–165)	0.618
Diastolic blood pressure, mm Hg (M, IQR)	90 (80–100)	90 (80–97)	0.916
Random blood sugar, mmol/L (M, IQR)	6.5 (5.5–7.8)	6.2 (5.3–7.3)	0.254
Weight, kg (M, IQR)	80 (67–90)	74 (66–85)	0.184
Onset time, min (M, IQR)	105 (80–150)	95 (65–140)	0.295
Door-to-needle time, min (M, IQR)	40 (30–60)	40 (30–60)	0.985
Stroke subtype, *n* (%)			
Atherothrombotic	26 (28.6)	56 (38.0)	0.133
Cardioembolic	29 (31.9)	43 (29.3)	0.669
Lacunar	1 (1.1)	4 (2.7)	0.396
Other known etiology	0 (0)	1 (0.7)	0.430
Unknown etiology	35 (38.4)	43 (29.3)	0.141
Drop-out patients, *n* (%)	7 (7.7)	13 (8.8)	0.756
Death	6 (6.6)	11 (7.5)	0.796
Neurosurgery	1 (1.1)	1 (0.7)	0.731
Severe medical condition	0 (0)	1 (0.7)	0.430
High HT risk (HTI 1–4), *n* (%)	57 (62.6)	80 (54.4)	0.213
HTI score (M, IQR)	1 (0–2)	1 (0–2)	0.655

**FIGURE 3:**
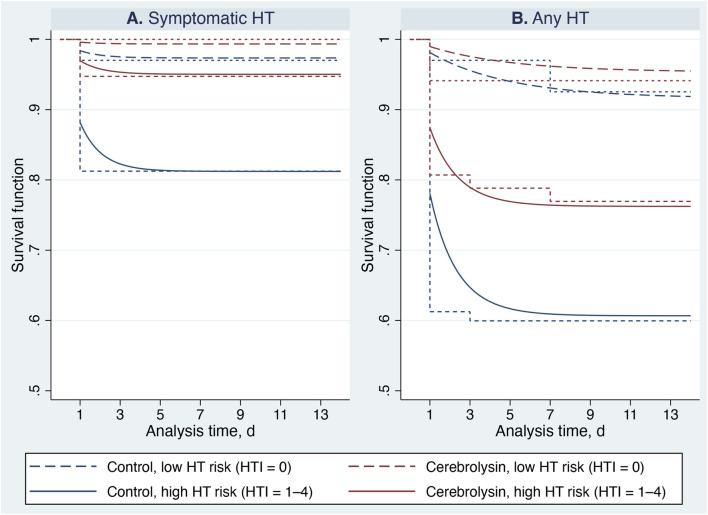
Non-parametric and parametric analysis. Standardized survival curves (dashed and solid lines) of the Gompertz model overlaid on Kaplan–Meier survival estimates (very-short-dashed and short-dashed lines represent low (HTI 0) and high (HTI 1–4) HT risk subgroups, respectively). Panel **(A)** Symptomatic hemorrhagic transformation. Panel **(B)** Any hemorrhagic transformation.

The nonparametric survival curves remained above the 50% survival probability due to the low incidence of HT, which precluded estimation of the median survival time (Table 2; Figure 3). In patients with low HT risk (HTI 0), Kaplan–Meier survival estimates did not differ between the Cerebrolysin and control groups (symptomatic HT: log-rank test, χ^2^ (1) = 1.03, *p* = 0.311; any HT: log-rank test, χ^2^ (1) = 0.07, *p* = 0.785; combined test, stochastic *p* = 0.9999, 95% CI 0.9992–0.9999). In contrast, a significant between-group difference was observed among high-risk patients (symptomatic HT: log-rank test, χ^2^ (1) = 5.27, *p* = 0.022; any HT: log-rank test, χ^2^ (1) = 4.45, *p* = 0.035; combined test, stochastic *p* = 0.021, 95% CI 0.017–0.025), indicating a beneficial effect of Cerebrolysin treatment in this cohort (Table 3; Figure 3).

**TABLE 2:** Summary of the survival data (*n* = 238).

Group	Symptomatic HT			Any HT		
	Time at risk, days	Incidence rate	Number of patients	Time at risk, days	Incidence rate	Number of patients
Low HT risk						
Control	908	0.002	67	887	0.006	67
Cerebrolysin	476	0	34	450	0.004	34
Total	1,384	0.001	101	1,337	0.005	101
High HT risk						
Control	853	0.018	80	645	0.050	80
Cerebrolysin	704	0.004	57	598	0.022	57
Total	1,557	0.012	137	1,243	0.036	137

**TABLE 3:** Number of patients at risk by analysis day (*n* = 238).

HT type	HT risk	Group	Analysis time, days													
			1	2	3	4	5	6	7	8	9	10	11	12	13	14
Symptomatic HT	Low	Control	67	65	65	65	65	65	65	65	65	65	64	64	64	64
		Cerebrolysin	34	34	34	34	34	34	34	34	34	34	34	34	34	34
	High	Control	80	63	63	62	61	60	60	59	59	59	57	57	57	56
		Cerebrolysin	57	51	50	50	50	50	50	50	50	50	49	49	49	49
Any HT	Low	Control	67	65	65	65	65	65	65	62	62	62	61	61	61	61
		Cerebrolysin	34	32	32	32	32	32	32	32	32	32	32	32	32	32
	High	Control	80	47	47	45	45	44	44	43	43	43	41	41	41	41
		Cerebrolysin	57	43	43	42	42	42	42	41	41	41	41	41	41	41

Baseline computed tomography (CT) was performed at enrollment (day 1). Scheduled follow-up CT scans occurred at 24 h (day 2), day 7, and day 14. HT-negative unscheduled CT scans were not documented in the parent trial. Attrition reflects all causes (HT events, death, or withdrawal) as recorded in the original dataset.

**FIGURE 4:**
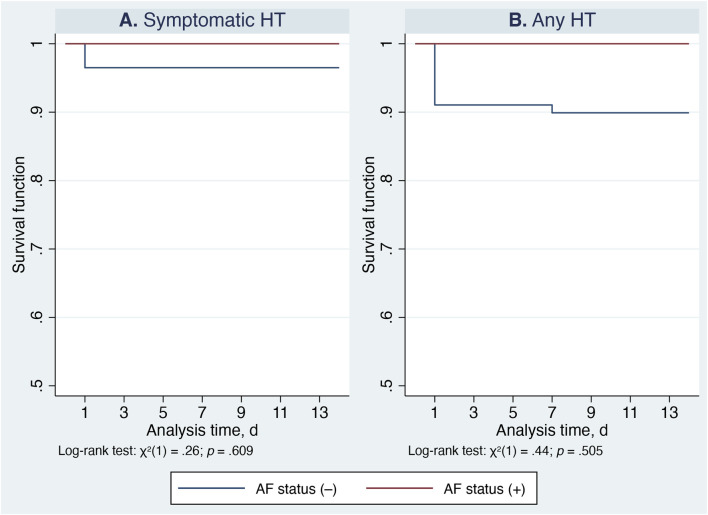
Kaplan–Meier survival estimates comparing patients with and without atrial fibrillation, adjusted for treatment group (Cerebrolysin vs. control) and predicted HT risk (low vs. high). Panel **(A)** Symptomatic hemorrhagic transformation. Panel **(B)** Any hemorrhagic transformation.

AF status did not meaningfully influence hemorrhagic outcomes. Kaplan–Meier curves adjusted for treatment group (Cerebrolysin vs. control) and predicted HT risk (low vs. high) showed no significant differences between patients with and without AF (symptomatic HT: χ^2^ (1) = 0.26, *p* = 0.609; any HT: χ^2^ (1) = 0.44, *p* = 0.505) (Figure 4). Because HT events within the AF subgroup were rare, subgroup-specific hazard modeling was not statistically reliable; therefore, the full dataset was retained for analyses related to anticoagulation timing.

**FIGURE 5:**
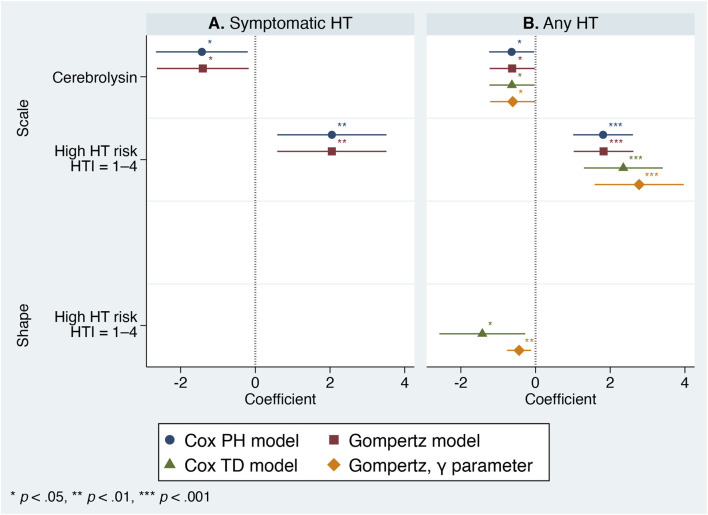
Comparison of semiparametric Cox proportional hazards and time-dependent models with parametric Gompertz models, estimated with and without the ancillary γ-parameter. Regression coefficients are shown with 95% confidence intervals. Panel **(A)** Symptomatic hemorrhagic transformation. Panel **(B)** Any hemorrhagic transformation.

Further refinement using the Cox PH model showed that treatment assignment (Cerebrolysin vs. control), adjusted for HT risk subgroup (low vs. high), significantly reduced the probability of HT, with an HR of 0.239 (95% CI 0.070–0.817, *p* = 0.022) for symptomatic HT and 0.527 (95% CI 0.289–0.962, *p* = 0.037) for any HT. However, the covariate violated the PH assumption (χ^2^ (2) = 11.19, *p* = 0.004) and interacted with time by altering the hazard function for any HT, although this violation was not observed for symptomatic HT (Figure 5).

**FIGURE 6:**
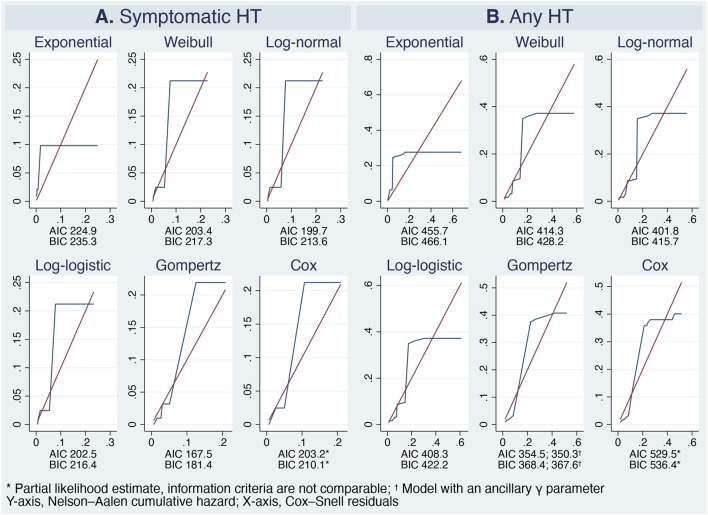
Model selection. The Nelson–Aalen cumulative hazard (navy) is overlaid on the Cox–Snell residuals (maroon). The Gompertz model is identified as the best fit based on the lowest Akaike (AIC) and Bayesian (BIC) information criteria values, as well as its similarity to the Cox proportional hazards model. Panel **(A)** Symptomatic hemorrhagic transformation. Panel **(B)** Any hemorrhagic transformation.

The data permitted fitting of the Royston–Parmar flexible parametric model with one degree of freedom for both the restricted cubic spline function and the TD effects, making it equivalent to the log-logistic, log-normal, or Weibull model depending on the chosen scale. Among all candidate distributions, the Gompertz model provided the best fit (Figure 6), indicating the hazard function declined exponentially over time—a pattern consistent with clinical observation.

Incorporation of the ancillary γ-parameter for the HT risk subgroup significantly modified the hazard shape for any HT and improved overall model performance (Figures 5, 6). Consequently, the standardized Gompertz survival curves closely overlapped the Kaplan–Meier estimates (Figure 3). Likewise, the Gompertz regression coefficients and goodness-of-fit statistics were comparable to those of the Cox model (Tables 4, 5; Figure 5).

**TABLE 4:** Post-estimation goodness-of-fit assessment.

Test	Symptomatic HT		Any HT		Comments
	Cox model	Gompertz model	Cox model	Gompertz model	
γ-shared frailty variance, θ	<0.001	<0.001	0.211	0.234*	Shared frailty across trial centers
LR test, ${\bar{\chi }}^{2}\left(1\right)$ , *p*-value	<0.001, 0.500	<0.001, >0.999	0.95, 0.165	1.09, 0.148*	Likelihood-ratio test of θ = 0; *p* > 0.05 indicates no center-level heterogeneity
γ-frailty variance, θ	—	<0.001	—	<0.001*	Unshared frailty
LR test, ${\bar{\chi }}^{2}\left(1\right)$ , *p*-value	—	<0.001, >0.999	—	<0.001, >0.999*	Likelihood-ratio test of θ = 0; *p* > 0.05 indicates no unobserved subject-level frailty
Link test, *p*-value	0.648	0.645	0.688	0.722	Test for model misspecification; *p* > 0.05 indicates no error
Grønnesby–Borgan test, $\chi^{2}\left(1\right)$ , *p*-value	0.282, 0.596	—	0.162, 0.687	—	Omnibus goodness-of-fit test for Cox PH model; *p* > 0.05 indicates adequate fit
Discrimination and explained variation	​	​	​	​	The higher values indicate better performance
Harrell’s *C*	0.758	0.758	0.735	0.735	​
Somers’ *D*	0.516	0.516	0.470	0.470	​
Royston–Sauerbrei *D*	2.095	2.079	1.507	1.845	​
Royston–Sauerbrei $R_{D}^{2}$	0.512	0.508	0.352	0.448	$R_{D}^{2}$ > 0.30 is acceptable

*The estimation settings did not permit fitting the Gompertz model with an ancillary parameter.

**TABLE 5:** Hausman specification test: pairwise comparison of coefficients related to the hazard scale.

Subject model	Object model			
	Cox model, symptomatic HT	Gompertz model, symptomatic HT	Cox model, any HT	Gompertz model, any HT
Cox model, symptomatic HT	—	χ^2^ (2) = 0.16; *p* = 0.922	χ^2^ (2) = 2.53; *p* = 0.283	χ^2^ (2) = 5.33; *p* = 0.070
Gompertz model, symptomatic HT	×	—	χ^2^ (2) = 2.39; *p* = 0.302	χ^2^ (2) = 5.18; *p* = 0.075
Cox model, any HT	×	×	—	χ^2^ (2) = 1.23; *p* = 0.540
Gompertz model, any HT	×	×	×	—

The symbol (×) indicates a violation of the test’s asymptotic assumptions. In each comparison, the subject model is the one being evaluated and initially assumed to be consistent and efficient. The object model is the model against which the subject is compared. When *p* ≥ 0.05, the object model is considered efficient, and the coefficient estimates of the two models are regarded as equivalent. When *p* < 0.05, the coefficients differ significantly, indicating that the object model is inconsistent and not suitable for further analysis.

The Gompertz ancillary γ-parameter was closely aligned with the Cox TD covariate for any HT. Accordingly, the influence of the predicted HT risk on any HT was strongest early after stroke onset and gradually diminished over time, whereas the treatment effect of Cerebrolysin remained constant. In contrast, neither ancillary parameters nor TD covariates were required for symptomatic HT, indicating that the effect of the included variables did not vary over time (Figure 5). The PH assumption may have been satisfied owing to the shorter time window in which symptomatic HT occurred (Figure 3), before the influence of predicted HT risk began to wane. The findings highlight the importance of HT risk stratification at admission and the early initiation of Cerebrolysin, which demonstrated a stable treatment effect even in patients with high and rapidly changing HT risk.

**FIGURE 7:**
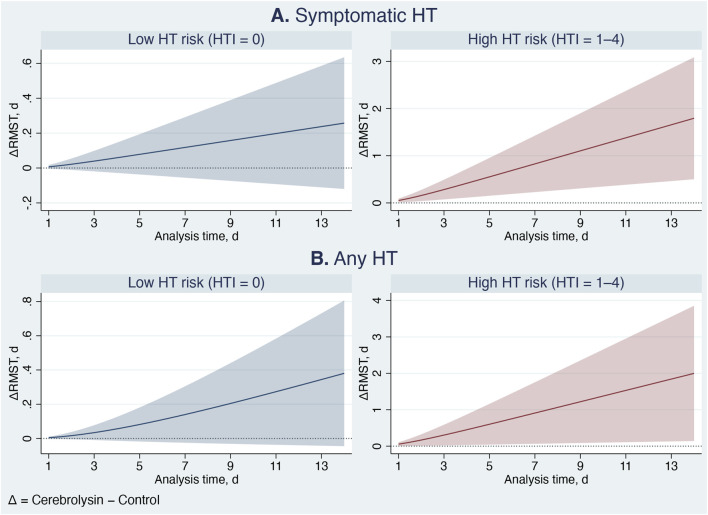
Cerebrolysin treatment effects: Differences in standardized restricted mean survival time (RMST) curves between Cerebrolysin and control groups, with 95% confidence intervals. Panel **(A)** Symptomatic hemorrhagic transformation. Panel **(B)** Any hemorrhagic transformation.

**FIGURE 8:**
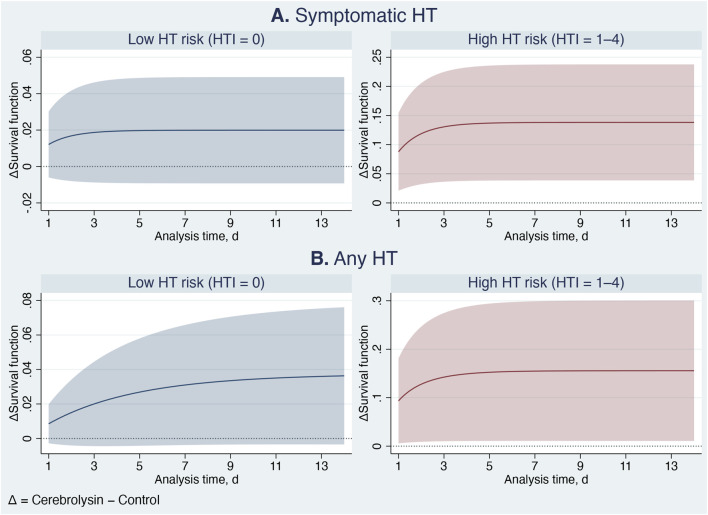
Cerebrolysin treatment effects: Differences in standardized survival curves (absolute risk reduction) between the Cerebrolysin and control groups, with 95% confidence intervals. Panel **(A)** Symptomatic hemorrhagic transformation. Panel **(B)** Any hemorrhagic transformation.

**FIGURE 9:**
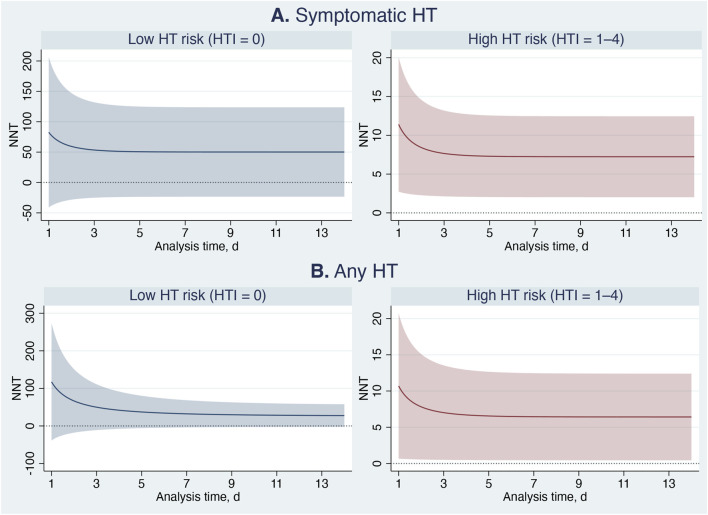
Cerebrolysin treatment effects: Number needed to treat (NNT) based on absolute risk reduction, with 95% confidence intervals. Panel **(A)** Symptomatic hemorrhagic transformation. Panel **(B)** Any hemorrhagic transformation.

The benefits of Cerebrolysin were even more pronounced in the parametric analysis (Table 6; Figures 7–9). Among patients with high HT risk, the population attributable fraction indicated that Cerebrolysin treatment could have prevented approximately 43% and 28% of symptomatic HT and any HT events, respectively. On average, Cerebrolysin delayed the occurrence of HT by nearly 2 days within the 14-day study period (Figure 7). The ARR increased during the first 3 days and then stabilized at about 15% (Figure 8), while the NNT plateaued at approximately seven (Figure 9). In contrast, the treatment conferred minimal benefit in patients with low HT risk.

**TABLE 6:** Parametric survival analysis: Cerebrolysin treatment effects adjusted for baseline HT risk.

Effect metrics	Symptomatic HT	Any HT
HR, mean (95% CI, *p*-value)	0.245 (0.072–0.837, 0.025)	0.543 (0.297–0.991, 0.047)
Romano–Wolf *p*-value	0.020	0.032
Population attributable fraction, mean (95% CI, *p*-value)		
All patients	0.466 (0.239–0.626, 0.001)	0.282 (0.052–0.457, 0.019)
High HT risk	0.427 (0.184–0.598, 0.002)	0.284 (0.063–0.452, 0.015)
Difference in RMST, mean (95% CI, *p*-value), days		
Low HT risk	0.3 (−0.1 … 0.6, 0.181)	0.4 (−0.1 … 0.8, 0.080)
High HT risk	1.8 (0.5–3.1, 0.007)	2.0 (0.1–3.9, 0.035)
ARR, mean (95% CI, *p*-value)		
Low HT risk	0.020 (−0.009 … 0.049, 0.181)	0.036 (−0.003 … 0.076, 0.074)
High HT risk	0.138 (0.039–0.238, 0.007)	0.156 (0.011–0.301, 0.035)
NNT, mean (95% CI, *p*-value)		
Low HT risk	50.2 (−23.3 … 123.7, 0.181)	27.5 (−2.6 … 57.7, 0.074)
High HT risk	7.2 (2.0–12.5, 0.007)	6.4 (0.5–12.4, 0.035)

The ARR, NNT, and RMST differences were calculated at the 14-day time horizon.

On the hazard scale, Gompertz regression coefficients did not differ significantly between the symptomatic HT and any HT models (Table 5). Accordingly, the symptomatic HT model was selected for further analysis to determine the optimal timing of anticoagulation resumption.

Sensitivity analysis across thresholds ranging from 2% to 13% of the NLHA peak demonstrated a smooth, monotonic shift in inception timing: higher thresholds produced earlier stabilization points without discontinuities, supporting stable and predictable system behavior (Table 7). Central inception estimates decreased gradually from 5.09 to 3.35 days, and internal uncertainty remained within a narrow ≈1-day band across all thresholds—an interval that is clinically negligible compared with real-world variability in anticoagulation initiation.

**TABLE 7:** Sensitivity analysis of NLHA thresholds.

Threshold, % of NLHA peak	Threshold, %/day	Inception point, lower bound, days	Inception point, mean, days	Central inception estimate, days	Internal uncertainty, days	Global representativeness, days
2	0.09	4.44	5.74	5.09	1.30	1.01
5	0.23	3.79	4.75	4.27	0.96	0.19
10	0.45	3.26	3.98	3.62	0.72	0.46
13	0.60	2.99	3.71	3.35	0.72	0.73

The 95%-CI lower-bound NLHA peak was 0.045%/day. The mean central inception estimate across all thresholds was ≈4.08 days.

**FIGURE 10:**
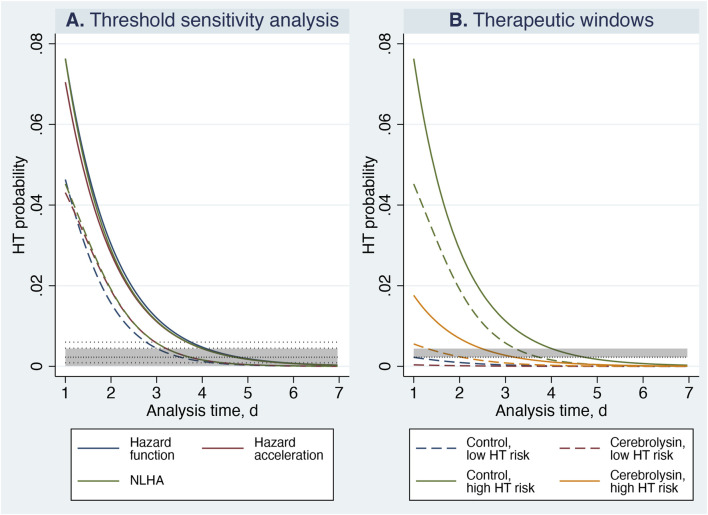
Timing of anticoagulation resumption based on NLHA dynamics. The analysis period is shown through day 7, beyond which hazard levels approached zero. Solid lines denote mean estimates and dashed lines denote 95%-CI lower bounds. Panel **(A)** Threshold sensitivity analysis in the most vulnerable subgroup (control patients with high HT risk, HTI 1–4). Horizontal dotted lines represent candidate NLHA thresholds of 0.09%, 0.23%, 0.45%, and 0.60% per day (2%, 5%, 10%, and 13% of the NLHA peak). The shaded band with a tight-dotted reference line corresponds to the 5% threshold (0.23%/day) and its 95% CI (0.01%–0.44%/day). Panel **(B)** Therapeutic windows for anticoagulation resumption. Standardized NLHA curves for treatment subgroups are displayed. The shaded band represents the established analytic threshold range (mean and upper 95% CI: 0.23%–0.44%/day). For clarity, mean curves for low HT-risk patients are not shown, as they closely approximate the lower-bound trajectories.

The 5% threshold (0.23%/day) emerged as the most representative cut-point. Its central inception estimate (4.27 days) lay closest to the overall mean across thresholds (≈4.08 days) and yielded the lowest global representativeness metric ( $D\left(\theta \right)$ = 0.19). At this level, the difference between lower-bound and mean trajectories (0.96 days) remained well under 1 day, indicating good internal coherence between the earliest and typical stabilization patterns. Notably, at the 5% threshold the hazard function, hazard acceleration, and NLHA curves demonstrated the closest convergence, indicating stabilization of hazard dynamics (Figure 10A).

By day 7, mean and 95%-CI lower-bound trajectories of these metrics had nearly reached zero and were essentially superimposed, demonstrating complete cessation of hazard evolution and confirming that HT risk had effectively plateaued. Collectively, these findings support the 5% threshold as the most balanced and data-driven approximation of the hemorrhagic–ischemic equilibrium. The narrow ≈1-day variation in inception estimates across thresholds further confirms the robustness of the sensitivity analysis.

The analytic threshold was 0.23%/day (95% CI 0.01%–0.44%/day). This interval overlapped with the empirically tested 2% threshold (0.09%/day), whereas the 10% and 13% cut-offs (0.45% and 0.60%/day) lay above the upper bound. Nonetheless, these higher thresholds shifted inception times by < 1 day, indicating no clinically meaningful divergence (Figure 10A). Taken together, these results confirm that the selected 5% cut-off lies centrally within the statistically supported range. For defining the therapeutic window for anticoagulation resumption, the mean and upper 95%-CI threshold values (0.23%–0.44%/day) were applied to ensure that inception points represent both the earliest defensible and the centrally representative stabilization boundaries (Figure 1).

Therapeutic windows for anticoagulation resumption varied substantially by baseline HT risk (Table 8; Figure 10B). In the low-risk subgroup (HTI 0), NLHA trajectories crossed the stabilization threshold within approximately 1–2 days in both treatment arms. Cerebrolysin-treated patients reached the threshold essentially by day 1, whereas control patients did so between days 1 and 2.5, indicating uniformly early stabilization regardless of treatment.

**TABLE 8:** Therapeutic windows for anticoagulation resumption (NLHA threshold 0.23%–0.44%/day).

Subgroup	Cerebrolysin	Control
Low HT risk, days	1.00–1.00	1.00–2.53
High HT risk, days	1.31–3.26	3.26–4.75

Values represent the earliest and typical stabilization times derived from the NLHA threshold range (0.23%–0.44%/day).

In contrast, high-risk patients (HTI 1–4) exhibited marked treatment-dependent differences. Cerebrolysin shortened the hazardous period by ≈1–2 days, with stabilization occurring between days 1.3 and 3.3, compared with days 3.3–4.8 in controls. The separation of the NLHA trajectories in this subgroup (Figure 10B) reflects earlier attenuation of HT risk under Cerebrolysin, resulting in an earlier therapeutic window for safe anticoagulation resumption.

**FIGURE 11:**
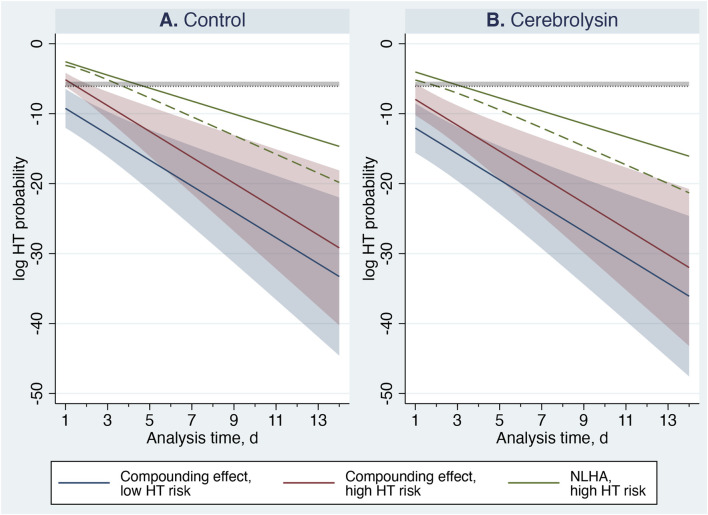
Compounding effect estimated using the quadratic hazard function ${h\left(t\right)}^{2}$ with 95% confidence intervals (CI). Curves are shown on a log scale for visualization. Solid lines denote mean estimates, and shaded regions represent 95% CIs. The green solid line shows the mean NLHA trajectory for high-risk patients (HTI 1–4), and the green dashed line depicting indicates its 95%-CI lower bound. The horizontal shaded band marks the analytic threshold range (mean and upper 95% CI: 0.23%–0.44%/day); compounding effect values above this band are considered clinically relevant. Panel **(A)** Control group. Panel **(B)** Cerebrolysin group.

The compounding effect—reflecting self-amplifying HT risk—was strongly modulated by baseline HT risk (Table 9; Figure 11). In high-risk controls, it peaked on day 1 and remained above threshold for ≈2 days, marking the most vulnerable segment of the hazardous period and highlighting the risk of premature anticoagulation. Cerebrolysin substantially attenuated this dynamic: the peak magnitude decreased by nearly 16-fold (0.583%/day vs. 0.035%/day), and the duration shortened to ≈1 day. This mitigation explains the earlier stabilization of hazard trajectories and the 1–2-day reduction in the hazardous period observed in high-risk patients. In low-risk patients, the compounding effect remained negligible under both conditions, consistent with low baseline hazard and rapid stabilization. Overall, these findings indicate that Cerebrolysin selectively dampens nonlinear escalation of HT risk in vulnerable patients, supporting a hazard-based, individualized approach to anticoagulation timing.

**TABLE 9:** Peak magnitude (day 1) and duration of the compounding effect.

Subgroup	Metric	Cerebrolysin	Control
Low HT risk	Magnitude (%/day), mean (95% CI)	<0.001 (<0.001–0.020)	0.010 (<0.001–0.157)
	Duration above threshold (days)	Below threshold	Below threshold
High HT risk	Magnitude (%/day), mean (95% CI)	0.035 (0.004–0.340)	0.583 (0.215–1.581)
	Duration above threshold (days)	1.00–1.23	1.84–2.34

Duration was calculated using the 95%-CI upper-bound trajectory of the quadratic hazard function. The stabilization threshold range was 0.23%–0.44%/day.

Based on these dynamics, anticoagulation may be safely resumed within ≈3 days with Cerebrolysin treatment and within ≈3–5 days with standard care in high-risk patients. For low-risk patients, anticoagulation may be reinitiated within ≈2 days regardless of treatment.

## Discussion

This *post hoc* secondary analysis evaluated the timing of anticoagulation resumption and the effect of Cerebrolysin on HT risk in patients with MCA stroke. By introducing hazard acceleration, NLHA, and the compounding effect as dynamic extensions of the hazard function, the study provides a more precise delineation of when HT risk stabilizes after IVT. Although the statistical modeling was rigorous, the central emphasis lies in how these dynamic risk metrics translate into clinically actionable guidance on anticoagulation timing.

Cerebrolysin’s impact on HT risk varied substantially according to patients’ baseline risk profile. Those with high HT risk (HTI 1–4) experienced the greatest benefit, showing both a marked attenuation of hazard magnitude and a shorter hazardous period. In contrast, low-risk patients (HTI 0) exhibited only minor improvements, consistent with their already low baseline hazard. This stratified treatment response aligns with earlier evidence of risk-dependent Cerebrolysin effects (Kalinin and Khasanova, 2024a; Kalinin and Khasanova, 2024b) and underscores that the timing of anticoagulation resumption should be tailored not only to baseline HT risk but also to its dynamic modification by treatment.

Anticoagulation with NOACs is central to secondary stroke prevention in patients with non-valvular AF, yet the optimal timing of resumption after ischemic stroke remains uncertain. Although guidelines still reference the empirical 1-3-6-12-day rule (Steffel et al., 2021), growing evidence from randomized and observational studies supports earlier initiation—within 48 h for minor-to-moderate strokes and within 4–5 days for larger infarcts—which appears safe and may reduce early ischemic recurrence (Seiffge et al., 2024; Palaiodimou et al., 2024; Alrohimi et al., 2023; Dehbi et al., 2025). The hazard-based time frame identified in our study is consistent with this evidence: lower predicted HT risk corresponds to mild or moderate strokes, whereas higher risk aligns with major infarcts, supporting the biological plausibility of the model and its agreement with prior observations (Kalinin et al., 2019; Kalinin and Khasanova, 2025).

Recent randomized trials—ELAN, OPTIMAS, TIMING, and START—and the CATALYST meta-analysis provide further context (Fischer et al., 2023; Dehbi et al., 2025; Werring et al., 2024; Oldgren et al., 2022; Warach et al., 2025). Across these studies, stroke severity was predominantly mild-to-moderate (median NIHSS 5), closely matching the clinical profile of our cohort. IVT use in CATALYST was modest (≈26–27%) due to its broad AF-stroke population, whereas our cohort consisted exclusively of IVT-treated MCA infarcts; nonetheless, the post-reperfusion HT risk dynamics are comparable. These trials consistently demonstrated that early NOAC initiation (typically ≤4 days) is safe and reduces early ischemic recurrence, with residual uncertainty restricted to the most severe strokes. The ATTUNE study (Sharobeam et al., 2024), conducted in a similarly mild-to-moderate population, likewise found fewer new ischemic lesions with NOAC initiation <4 days.

Definitions of “early” and “late” anticoagulation varied substantially across the trials, complicating direct comparisons. In CATALYST, early NOAC initiation was defined as initiation ≤4 days of stroke onset and later initiation as ≥5 days (Dehbi et al., 2025), while other trials linked timing to infarct size rather than explicit hazard dynamics. In contrast, our framework defines early and late initiation based on individualized baseline HT risk rather than fixed calendar-day cutoffs. Within this model, early initiation corresponds to ≤48 h for patients with low predicted HT risk and approximately 3–5 days for those with high risk, whereas initiation beyond 5 days consistently falls into the late window. This risk-grounded approach accommodates patient-level heterogeneity and provides a physiologic rationale for timing categories that differs from—but complements—the empiric definitions used in randomized trials.

Most trials did not report stroke location, limiting anterior–posterior comparisons. ATTUNE was the only study to provide such data (82% anterior; 18% posterior) (Sharobeam et al., 2024), consistent with the expected predominance of anterior-circulation events in AF-related stroke. Our cohort—exclusively MCA anterior-circulation infarcts treated with IVT—therefore represents a more homogeneous, reperfusion-treated population.

Although anticoagulation timing is most clinically relevant in patients with AF, an AF-specific sub-analysis was not performed because the number of HT events—particularly symptomatic HT—was insufficient for stable hazard estimation. Importantly, AF is incorporated into the HTI score (Kalinin et al., 2017), so patients with similar HTI values share comparable predicted HT trajectories regardless of AF status, preserving the model’s applicability to AF populations despite their smaller representation in our cohort. The model’s assumptions, outlined in the *Strengths, limitations, and future research directions* section, are appropriate for NOAC therapy and support cautious interpretation of the hazard-stabilization point. Within this framework, the estimated therapeutic window (approximately days 3–5 under standard care) complements conventional AF practice—typically days 3–6 under the 1-3-6-12 rule—by offering a physiologic, individualized rationale for earlier initiation when HT risk has already declined.

In patients with acute stroke and AF, the daily risk of early ischemic recurrence ranges from 0.1% to 1.3% (Paciaroni et al., 2016) and may be even higher in those previously treated with anticoagulants (Tanaka et al., 2020; Seiffge et al., 2020). The validity of the established HT-stabilization threshold (0.23%–0.44%/day) was supported by the consistent convergence of the hazard function, hazard acceleration, and NLHA curves at the inception points, indicating that HT risk had fallen to clinically negligible levels, comparable to the competing ischemic risk. Our prior study (Kalinin and Khasanova, 2025) identified a higher threshold of 0.6%/day in patients with very high hemorrhagic susceptibility (HTI 5–8). Notably, applying thresholds across the full 0.09%–0.6%/day range produced only modest variation in inception points, demonstrating the robustness of the hazard-dynamics model. Taken together, the derived threshold range provides conservative and clinically coherent bounds for guiding the safe timing of anticoagulation resumption.

Recent clinical trials and pooled analyses suggest that initiating NOAC therapy within 48 h after reperfusion therapy does not increase the risk of HT (Sharobeam et al., 2024; Alrohimi et al., 2023; Suzuki et al., 2017; Kimura et al., 2022). In parallel, accumulating evidence from observational studies and meta-analyses indicates that patients who undergo IVT while on NOACs do not experience a substantial rise in bleeding events (Meinel et al., 2023; Ghannam et al., 2023; Behnoush et al., 2023). These findings support our hazard-based framework, reinforcing its applicability to NOACs and underscoring the importance of early anticoagulation resumption, particularly in patients with prior anticoagulant exposure. In this context, Cerebrolysin may further facilitate earlier and safer reinitiation of anticoagulation, potentially improving outcomes for high-risk patients. However, it should be noted that our trial excluded individuals who had taken NOACs within 48 h before the index stroke, in accordance with contemporary clinical guidelines (Berge et al., 2021).

In relation to HT, the compounding effect was clearly demonstrated in stroke patients, with its temporal characteristics comprehensively delineated. This self-amplifying escalation of instantaneous HT risk plays a pivotal role in determining the optimal timing of anticoagulation resumption. The greater the HT risk at the beginning, the higher the magnitude of the compounding effect, and the longer the delay required before anticoagulation can be safely reinitiated. These dynamics underscore the need for an individualized, hazard-based approach to treatment planning, particularly in high-risk patients.

Premature resumption of anticoagulation during the hazardous period may further potentiate the compounding effect, markedly increasing the likelihood of HT. Accurate estimation of early HT risk is therefore essential for therapeutic optimization. Moreover, early administration of treatments that mitigate HT risk can attenuate the compounding effect and prevent its subsequent amplification. In this context, concomitant use of Cerebrolysin with IVT proved beneficial in high-risk patients, diminishing the compounding effect, reducing overall HT risk, and enabling earlier and safer anticoagulation resumption.

The observed benefit likely stems from Cerebrolysin’s modulation of key pathophysiological mechanisms underlying HT. Focal ischemia followed by reperfusion injury triggers a cascade of deleterious events, including excitotoxicity, blood–brain barrier (BBB) disruption, and neuroinflammation—processes exacerbated by alteplase and strongly implicated in HT development (Hong et al., 2021; Spronk et al., 2021; Wang et al., 2004; Kelly et al., 2006). Evidence from both clinical and experimental studies indicates that Cerebrolysin stabilizes the BBB, preserves neurovascular unit integrity, and attenuates inflammatory responses in ischemic tissue (Khasanova and Kalinin, 2023a; Zhang et al., 2010; Teng et al., 2021; Veinbergs et al., 2000; Guan et al., 2019). By mitigating these pathophysiological pathways, Cerebrolysin may slow the evolution of HT and lower its overall incidence.

Several adjuvant cytoprotective agents have been investigated in combination with IVT in patients with acute ischemic stroke (Dammavalam et al., 2024; Mazighi et al., 2024). However, large phase III trials are still needed to confirm the encouraging results observed in earlier studies (Otsu et al., 2020). The ESCAPE-NA1 trial—a pivotal phase III study of the cytoprotective peptide nerinetide—failed to demonstrate improved functional outcomes, likely due to pharmacological interactions with alteplase. Moreover, nerinetide did not affect the incidence of HT in either treatment groups (Hill et al., 2020). In this context, Cerebrolysin may represent a viable alternative to nerinetide and other candidates, given its ability to counteract alteplase-related adverse effects (Teng et al., 2021), well-established safety profile, and long-lasting clinical use worldwide (Strilciuc et al., 2021).

Contemporary clinical guidelines and recent studies (Steffel et al., 2021; Kimura et al., 2022) rely mainly on stroke severity assessed by the NIHSS or infarct volume on brain imaging to guide the timing of anticoagulation resumption. Although straightforward, this approach overlooks the multifactorial nature of HT. Using composite scores for HT prediction is therefore more rational. Several tools have been proposed; while many show good reliability and include similar predictors, only the HTI score accounts for the vascular territory of the infarct. Our previous studies demonstrated that the HTI score has superior predictive performance compared with other tools (Kalinin and Khasanova, 2024a; Kalinin and Khasanova, 2024b; Kalinin et al., 2017; Kalinin et al., 2019). Given the well-established differences between anterior and posterior circulation strokes, this may explain its stronger accuracy in patients with MCA infarction.

Since early reports on IVT-related symptomatic HT (The NINDS t-PA Stroke Study Group, 1997), most studies have analyzed the follow-up period as a single interval rather than as a continuum of instantaneous events, presenting results mainly as HT odds ratios and descriptive statistics. To our knowledge, relatively few authors have applied nonparametric and semiparametric survival analysis on this topic (Kimura et al., 2022; Muscari et al., 2020). In contrast, we employed more advanced approaches to characterize hazard dynamics in stroke patients, and the implications of our findings may provide a theoretical framework for future research.

The emergence of next-generation intravenous thrombolytics and advances in endovascular thrombectomy (EVT) introduce additional considerations for HT risk assessment and anticoagulation timing (Seiffge et al., 2024; Dammavalam et al., 2024). In the CEREHETIS trial, patients with very high predicted HT risk (HTI 5–8) were excluded because large infarct cores or severe strokes made them ineligible for IVT (Khasanova and Kalinin, 2023a; Khasanova and Kalinin, 2023b). Some may have been potential EVT candidates, although the implications for HT dynamics in this subgroup remain uncertain. Meta-analyses of EVT in large-core infarction show improved functional outcomes compared with best medical treatment but consistently report higher HT rates (Morsi et al., 2024), underscoring the need for careful risk–benefit evaluation in population with substantial baseline risk. A preliminary study of Cerebrolysin suggested potential safety in patients with severe stroke and futile recanalization after IVT (Poljakovic et al., 2021), and exploratory reports of its use alongside EVT have described improved outcomes and reduced HT incidence (ElBassiouny et al., 2025; Staszewski et al., 2025). However, these findings remain hypothesis-generating and require prospective validation. At present, the applicability of our hazard-based framework to non-MCA, EVT-treated, or large-core populations is uncertain and should not be inferred from the current dataset.

### Strengths, limitations, and future research directions

A key strength of this study is the detailed stratification of HT risk, enabling targeted evaluation of Cerebrolysin’s effects and supporting a personalized approach to anticoagulation timing. Methodological rigor is reinforced by the use of complementary hazard-dynamics metrics, which capture temporal features of HT risk not addressed by conventional analyses. Clinically, the findings offer a structured, physiology-based framework for timing anticoagulation after IVT-treated MCA stroke and are broadly consistent with emerging trial evidence on early NOAC initiation.

Several limitations warrant consideration. The timing estimates arise from *post-hoc* hazard-dynamics modeling rather than prospective trials, limiting direct clinical application. The analytical framework relies on assumptions—such as monotonic hazard decline and the non-interference of NOACs with HT trajectories—which were satisfied in this dataset but require external validation. Second, the focus on MCA infarctions restricts generalizability; posterior circulation strokes and other vascular territories may exhibit different HT profiles. The cohort reflects IVT-eligible MCA strokes from the parent trial. Patients with HTI >4 were absent because they did not meet IVT eligibility, not due to deliberate exclusion; thus, the findings apply only to patients with HTI 0–4. While stratified analyses (HTI 0 vs. HTI 1–4) showed consistent hazard patterns, some strata were modest in size, reducing statistical precision. Finally, as a secondary *post-hoc* analysis, the study is susceptible to residual confounding and spurious associations.

Future research should evaluate Cerebrolysin across a broader spectrum of stroke severities, comorbidities, and vascular territories through prospective, real-world studies. Dedicated investigations are needed to determine whether hazard-stabilization principles generalize to higher-risk populations, including non-MCA strokes and advanced reperfusion strategies. Further exploration of Cerebrolysin in combination with next-generation thrombolytics (e.g., tenecteplase) and modern EVT techniques is warranted. Studies examining co-administration with dual antiplatelet therapy or NOACs will clarify safety and pharmacologic interactions. Investigation into adjunctive use in extended reperfusion windows may determine whether Cerebrolysin enhances cytoprotection and reduces HT risk when reperfusion is delayed. Long-term outcome and safety data will ultimately be necessary to refine treatment algorithms and optimize acute stroke care.

## Conclusion

This hazard-based analysis suggests that the timing of anticoagulation resumption after IVT-treated MCA stroke depends on the admission HTI-defined risk profile. In low-risk patients (HTI 0), NOAC therapy appeared safe to restart within 48 h under both Cerebrolysin and standard care. In higher-risk but still IVT-eligible patients (HTI 1–4), Cerebrolysin was associated with earlier stabilization of HT hazard, indicating the possibility of advancing anticoagulation to approximately the first 3 days, compared with about 3–5 days under standard care.

These findings—derived from *post-hoc* modeling in MCA strokes with HTI 0–4—are preliminary and should not be extrapolated to other vascular territories or EVT-treated populations. They provide hypothesis-generating evidence that Cerebrolysin may allow earlier NOAC resumption by roughly 1–2 days in selected high-risk patients, warranting prospective validation.

## Clinical perspective

What is new?

- We found that hemorrhagic risk after MCA stroke settles earlier than traditionally assumed.
- Patients receiving Cerebrolysin reached a stable, lower-risk period about 1–2 days sooner than those on standard care.

What are the clinical implications?

- These findings suggest that anticoagulation might be started earlier in some patients, especially when Cerebrolysin is used.
- This is exploratory evidence only, and prospective studies are needed before changing practice.

## Data Availability

The original contributions presented in the study are included in the article/Supplementary Material, further inquiries can be directed to the corresponding author.

## Supplementary material

SUPPLEMENTARY DATA SHEET 1 Raw data.

SUPPLEMENTARY DATA SHEET 2 Stata code.
