# Supplementary material for: Cerebrolysin, hemorrhagic transformation, and anticoagulation timing after reperfusion therapy in stroke: post hoc secondary analysis of the CEREHETIS trial
Source: Front Pharmacol. 2026 Jan 7;16:1725255. doi: 10.3389/fphar.2025.1725255 (PMC12865818; doi:10.3389/fphar.2025.1725255)
Supplement: Supplementary file 1 [file DataSheet2.pdf]

```
* Supplementary Material: Stata Code

* Cerebrolysin, Hemorrhagic Transformation, and Anticoagulation Timing after Reperfusion Therapy in Stroke: Secondary Analysis of the CEREHETIS Trial
* Mikhail N. Kalinin and Dina R. Khasanova

* 1. Department of Neurology, Kazan State Medical University, Kazan, Russia
* 2. Department of Neurology, Interregional Clinical Diagnostic Center, 12A Karbyshev str., Kazan, 420101, Russia

//requried packages to install:
* stctest
* stpm3
* stpm2
* str2d
* somersd
* stcoxgof
* rwolf2
* coefplot
* standsurv
* punafcc
* grc1leg


//generating analysis time for anyht:
gen any_time = HTday if AnyHT==1
replace any_time = ExitDay if any_time==.
replace any_time = 14 if any_time==.
replace any_time = 14 if any_time>14

//gen analysis time for sht:
gen sht_time = HTday if symHT==1
replace sht_time = ExitDay if sht_time==.
replace sht_time = 14 if sht_time==.
replace sht_time = 14 if sht_time>14

//generating ht risk group (low risk = HTI=0, high risk = HTI >0)
gen ht_risk = 0 if HTI==0
replace ht_risk = 1 if HTI>0
label define ht_risk 0 "HTI = 0" 1 "HTI > 0"
label values ht_risk ht_risk

//nonparametric analysis:
*any ht
qui: stset any_time, failure(AnyHT==1)
by Group ht_risk, sort : stdescribe
by ht_risk, sort : stsum, by(Group)
** equality of survival curves - logrank test:
sts test Group, logrank strata(ht_risk) detail
** equality of survival curves - combine test:
```

```
set seed 123
stctest ps Group if ht_risk==1,  nperm(5000)
stctest ps Group if ht_risk==0,  nperm(5000)
*symptomatic ht
stset, clear
qui: stset sht_time, failure(symHT==1)
by Group ht_risk, sort : stdescribe
by ht_risk, sort : stsum, by(Group)
** equality of survival curves - logrank test:
sts test Group, logrank strata(ht_risk) detail

//semiparametric analysis: Cox model
*Cox PH model:
stset, clear
qui: stset any_time, failure(AnyHT==1)
stcox Group ht_risk, nolog efron
estat phtest
stphplot, by(Group) adjustfor(ht_risk)
stset, clear
qui: stset sht_time, failure(symHT==1)
stcox Group ht_risk, nolog efron
estat phtest
stphplot, by(Group) adjustfor(ht_risk)

//parametric analysis:
*chooosing model - calculating AIC and BIC:
**for any ht:
qui: stset any_time, failure(AnyHT==1)
foreach model in exponential weibull gompertz lognormal loglogistic {
qui: streg Group ht_risk, dist(`model')
estimates store `model'
}
estimates stats _all
drop _est*
foreach scale in logcumhazard logodds probit {
display _n "Scale = `scale'"
forvalues j = 1/1 {
qui: stpm3 Group ht_risk, df(`j') scale(`scale')
display "df = `j', AIC = " %7.2f e(AIC) " BIC = " %7.2f e(BIC)
}
}
**for sympt ht:
stset, clear
qui: stset sht_time, failure(symHT==1)
foreach model in exponential weibull gompertz lognormal loglogistic {
qui: streg Group ht_risk, dist(`model')
estimates store `model'
}
estimates stats _all
drop _est*
```

```
foreach scale in logcumhazard logodds probit {
display _n "Scale = `scale'"
forvalues j = 1/1 {
quietly xi: stpm3 Group ht_risk, df(`j') scale(`scale')
display "df = `j', AIC = " %7.2f e(AIC) " BIC = " %7.2f e(BIC)
}
}
drop _*
stset, clear

//Figure 6 cox-snell residuals combo:
qui: stset sht_time, failure(symHT==1)
foreach model in exponential weibull gompertz lognormal loglogistic {
qui: streg Group ht_risk, dist(`model')
estimates store `model'
predict cs, csnell
qui: stset cs, failure(symHT==1)
qui: sts generate H = na
estat ic
mat es_ic = r(S)
local aic: display %4.1f es_ic[1,5]
local bic: display %4.1f es_ic[1,6]
line H cs cs, sort title("`model'") xtitle("AIC `aic'" "BIC `bic'") name(`model', replace) nodraw
drop H cs
stset, clear
qui: stset sht_time, failure(symHT ==1)
}
qui: stcox Group ht_risk, nohr nolog efron mgale(mg)
est store cox
predict cs, csnell
qui: stset cs, failure(symHT==1)
qui: sts generate H = na
estat ic
mat es_ic = r(S)
local aic: display %4.1f es_ic[1,5]
local bic: display %4.1f es_ic[1,6]
line H cs cs, sort title("cox") xtitle("AIC `aic'" "BIC `bic'") name(cox, replace) nodraw
drop H cs mg
estimates stats _all
drop _est*
grc1leg exponential weibull lognormal loglogistic gompertz cox, title("{bf:A.} Symptomatic HT") name(g1, replace) legendfrom(cox)
stset, clear
qui: stset any_time, failure(AnyHT==1)
foreach model in exponential weibull gompertz lognormal loglogistic {
qui: streg Group ht_risk, dist(`model')
estimates store `model'
predict cs, csnell
```

```
qui: stset cs, failure(AnyHT==1)
qui: sts generate H = na
estat ic
mat es_ic = r(S)
local aic: display %4.1f es_ic[1,5]
local bic: display %4.1f es_ic[1,6]
line H cs cs, sort title("`model'") xtitle("AIC `aic'" "BIC `bic'") name(`model', replace) nodraw
drop H cs
stset, clear
qui: stset any_time, failure(AnyHT==1)
}
qui: stcox Group ht_risk, nohr nolog efron mgale(mg)
est store cox
predict cs, csnell
qui: stset cs, failure(AnyHT==1)
qui: sts generate H = na
estat ic
mat es_ic = r(S)
local aic: display %4.1f es_ic[1,5]
local bic: display %4.1f es_ic[1,6]
line H cs cs, sort title("cox") xtitle("AIC `aic'" "BIC `bic'") name(cox, replace) nodraw
drop H cs mg
stset, clear
qui: stset any_time, failure(AnyHT==1)
qui: streg Group ht_risk, distribution(gompertz) ancillary (ht_risk) nolog
est store gompertz_ax
estimates stats _all
drop _est*
grc1leg exponential weibull lognormal loglogistic gompertz cox, title("{bf:B.} Any HT") name(g2, replace) legendfrom(cox)
grc1leg g1 g2, legendfrom(g1)

//Table 4. cox and gompertz models: goodness-of-fit statictics:
* shared gamma frailty cox model, gompertz, cox td models, group shared frailty - center
stset, clear
qui: stset sht_time, failure(symHT==1)
stcox Group ht_risk, shared(Center) efron nolog nohr
streg Group ht_risk, distribution(gompertz) frailty(gamma) shared(Center) nolog nohr
stset, clear
qui: stset any_time, failure(AnyHT==1)
stcox Group ht_risk, shared(Center) efron nolog nohr
streg Group ht_risk, distribution(gompertz) frailty(gamma) shared(Center) nolog nohr
stcox Group ht_risk, tvc(Group ht_risk) texp(ln(_t)) shared(Center) efron nolog nohr
stcox Group ht_risk, tvc(ht_risk) texp(ln(_t)) shared(Center) efron nolog nohr
```

```
* unshared gamma frailty cox model, gompertz, cox td models:
stset, clear
qui: stset sht_time, failure(symHT==1)
streg Group ht_risk, distribution(gompertz) frailty(gamma)  nolog nohr
stset, clear
qui: stset any_time, failure(AnyHT==1)
streg Group ht_risk, distribution(gompertz) frailty(gamma) nolog nohr
//diagnostic of cox and gompertz models:
* sht:
stset, clear
qui: stset sht_time, failure(symHT==1)
str2d: stcox Group ht_risk, nolog efron
str2d: streg Group ht_risk, distribution(gompertz) nolog
stcox Group ht_risk, nolog efron
predict hr, hr
generate invhr=1/hr
generate censind=1-_d if _st==1
somersd _t invhr if _st==1, cenind(censind) tdist transf(c)
somersd _t invhr if _st==1, cenind(censind) tdist transf(z)
drop hr invhr
streg Group ht_risk, distribution(gompertz) nolog
predict hr, hr
generate invhr=1/hr
somersd _t invhr if _st==1, cenind(censind) tdist transf(c)
somersd _t invhr if _st==1, cenind(censind) tdist transf(z)
drop censind hr invhr
** specificatijon error
stcox Group ht_risk,  efron nolog nohr
linktest, efron nolog
streg Group ht_risk, distribution(gompertz) nohr nolog
linktest, dist(gompertz) nolog nohr
* anyht:
stset, clear
qui: stset any_time, failure(AnyHT==1)
str2d: stcox Group ht_risk, nolog efron
str2d: streg Group ht_risk, distribution(gompertz) ancillary (ht_risk) nolog
stcox Group ht_risk, nolog efron
predict hr, hr
generate invhr=1/hr
generate censind=1-_d if _st==1
somersd _t invhr if _st==1, cenind(censind) tdist transf(c)
somersd _t invhr if _st==1, cenind(censind) tdist transf(z)
drop hr invhr
streg Group ht_risk, distribution(gompertz) ancillary (ht_risk) nolog
predict hr, hr
generate invhr=1/hr
somersd _t invhr if _st==1, cenind(censind) tdist transf(c)
somersd _t invhr if _st==1, cenind(censind) tdist transf(z)
```

```
drop censind hr invhr
stcox Group ht_risk,  efron nolog nohr
linktest, efron nolog
streg Group ht_risk, distribution(gompertz) ancillary (ht_risk) nolog
linktest, dist(gompertz) ancillary (ht_risk)  nolog nohr
* goodness-of-fit Groennesby and Borgan test
stset, clear
qui: stset sht_time, failure(symHT==1)
qui: stcox Group ht_risk, nohr nolog efron mgale(mg)
stcoxgof
drop mg
stset, clear
qui: stset any_time, failure(AnyHT==1)
qui: stcox Group ht_risk, nohr nolog efron mgale(mg)
stcoxgof
drop mg

//Figure 5: coefplot for sht and anyht tvc and gamma gompertz:
qui: stset sht_time, failure(symHT==1)
qui: stcox Group ht_risk, nolog nohr efron
est store cox
qui: streg Group ht_risk, distribution(gompertz) nohr nolog
est store gom
stset, clear
qui: stset any_time, failure(AnyHT==1)
qui: stcox Group ht_risk, nohr nolog efron
est store acox
qui: stcox Group ht_risk, tvc(ht_risk) texp(ln(_t)) nohr efron nolog
est store tdcox
qui: streg Group ht_risk, distribution(gompertz) nohr nolog
est store agom
qui: streg Group ht_risk, distribution(gompertz) ancillary (ht_risk) nolog
est store ggom
coefplot (cox, eqrename(_ = main)) (gom, eqrename(_t = main)) || ( acox, eqrename(_ = main)) (agom, eqrename(_t = main)) (tdcox, eqrename(tvc = gamma) ) (ggom, eqrename(_t = main)), xline(0) keep (*:) drop(_cons gamma) nolabel mlabposition(2)  mlabel(cond(@pval<.001, "***", cond(@pval<.01, "**",  cond(@pval<.05, "*",  "")))) note("* {it:p} < .05, ** {it:p} < .01, *** {it:p} < .001")
drop _est_cox _est_gom _est_acox _est_agom _est_tdcox _est_ggom
stset, clear

//Figures 3:
range temptime 1 14
* mata function for NNT
mata
function calcNNT(at) {
return(1/(at[2]-at[1]))
}
```

```
end
//predictions, standardized curves for sht:
qui: stset sht_time, failure(symHT==1)
qui: streg Group ht_risk, distribution(gompertz) nolog
standsurv, at1(Group 0 ht_risk 0) at2(Group 1 ht_risk 0) ci timevar(temptime) survival userfunction(calcNNT) userfunctionvar(nnt21s)
drop _at*
standsurv, at1(Group 0 ht_risk 1) at2(Group 1 ht_risk 1) ci timevar(temptime) survival userfunction(calcNNT) userfunctionvar(nnt43s)
drop _at*
standsurv, atvar(s1 s2) at1(Group 0 ht_risk 0) at2(Group 1 ht_risk 0) timevar(temptime) survival  ci contrastvar(sdiff21) contrast(difference)
standsurv, atvar(s3 s4) at1(Group 0 ht_risk 1) at2(Group 1 ht_risk 1) timevar(temptime) survival  ci contrastvar(sdiff43) contrast(difference)
standsurv, atvar(h1 h2) at1(Group 0 ht_risk 0) at2(Group 1 ht_risk 0) timevar(temptime) hazard ci contrastvar(hdiff21) contrast(difference)
standsurv, atvar(h3 h4) at1(Group 0 ht_risk 1) at2(Group 1 ht_risk 1) timevar(temptime) hazard ci contrastvar(hdiff43) contrast(difference)
standsurv, atvar(rmst1 rmst2) at1(Group 0 ht_risk 0) at2(Group 1 ht_risk 0) timevar(temptime) rmst ci contrastvar(rmst_diff21) contrast(difference)
standsurv, atvar(rmst3 rmst4) at1(Group 0 ht_risk 1) at2(Group 1 ht_risk 1) timevar(temptime) rmst ci contrastvar(rmst_diff43) contrast(difference)
//Figure 3A:
sts graph, by(Group ht_risk) survival addplot (line s1 s3 s2 s4 temptime, sort) legend(off) name(g1, replace) nodraw
//predictions, standardized curves for any ht:
stset, clear
qui: stset any_time, failure(AnyHT==1)
qui: streg Group ht_risk, distribution(gompertz) ancillary (ht_risk) nolog
standsurv, at1(Group 0 ht_risk 0) at2(Group 1 ht_risk 0) ci timevar(temptime) survival userfunction(calcNNT) userfunctionvar(nnt21a)
drop _at*
standsurv, at1(Group 0 ht_risk 1) at2(Group 1 ht_risk 1) ci timevar(temptime) survival userfunction(calcNNT) userfunctionvar(nnt43a)
drop _at*
standsurv, atvar(sa1 sa2) at1(Group 0 ht_risk 0) at2(Group 1 ht_risk 0) timevar(temptime) survival  ci contrastvar(sadiff21) contrast(difference)
standsurv, atvar(sa3 sa4) at1(Group 0 ht_risk 1) at2(Group 1 ht_risk 1) timevar(temptime) survival  ci contrastvar(sadiff43) contrast(difference)
standsurv, atvar(ha1 ha2) at1(Group 0 ht_risk 0) at2(Group 1 ht_risk 0) timevar(temptime) hazard ci contrastvar(hadiff21) contrast(difference)
```

```
standsurv, atvar(ha1 ha2) at1(Group 0 ht_risk 0) at2(Group 1 ht_risk 0) timevar(temptime) hazard ci contrastvar(hadiff21) contrast(difference)
standsurv, atvar(ha3 ha4) at1(Group 0 ht_risk 1) at2(Group 1 ht_risk 1) timevar(temptime) hazard ci contrastvar(hadiff43) contrast(difference)
standsurv, atvar(rmsta1 rmsta2) at1(Group 0 ht_risk 0) at2(Group 1 ht_risk 0) timevar(temptime) rmst ci contrastvar(rmsta_diff21) contrast(difference)
standsurv, atvar(rmsta3 rmsta4) at1(Group 0 ht_risk 1) at2(Group 1 ht_risk 1) timevar(temptime) rmst ci contrastvar(rmsta_diff43) contrast(difference)
//Figure 3B:
sts graph, by(Group ht_risk) survival addplot (line sa1 sa3 sa2 sa4 temptime, sort) legend(off) name(g2, replace) nodraw
//Figure 3 combo:
grc1leg g1 g2, cols(2) legendfrom(g1)
mata: mata clear

//figure 7 - difference in rmst:
twoway (rarea rmst_diff21_lci rmst_diff21_uci temp, sort) (line rmst_diff21 temptime, sort),legend(off) yline(0) name(g1, replace) nodraw
twoway (rarea rmst_diff43_lci rmst_diff43_uci temp, sort) (line rmst_diff43 temptime, sort), legend(off) yline(0) name(g2, replace) nodraw
twoway (rarea rmsta_diff21_lci rmsta_diff21_uci temp, sort) (line rmsta_diff21 temptime, sort), legend(off) yline(0) name(g3, replace) nodraw
twoway (rarea rmsta_diff43_lci rmsta_diff43_uci temp, sort) (line rmsta_diff43 temptime, sort), legend(off) yline(0) name(g4, replace) nodraw
graph combine g1 g2, name(g5, replace) nodraw
graph combine g3 g4, name(g6, replace) nodraw
graph combine g5 g6, rows(2)

//figure 8 difference in survaval (arr):
twoway (rarea sdiff21_lci sdiff21_uci temp, sort) (line sdiff21 temptime, sort), legend(off) yline(0) name(g1, replace) nodraw
twoway (rarea sdiff43_lci sdiff43_uci temp, sort) (line sdiff43 temptime, sort), legend(off) yline(0) name(g2, replace) nodraw
twoway (rarea sadiff21_lci sadiff21_uci temp, sort) (line sadiff21 temptime, sort), legend(off) yline(0) name(g3, replace) nodraw
twoway (rarea sadiff43_lci sadiff43_uci temp, sort) (line sadiff43 temptime, sort), legend(off) yline(0) name(g4, replace) nodraw
graph combine g1 g2, name(g5, replace) nodraw
graph combine g3 g4, name(g6, replace) nodraw
graph combine g5 g6, rows(2)

//figure 9 NNT
twoway (rarea nnt21s_lci nnt21s_uci temp, sort) (line nnt21s temptime, sort), legend(off) yline(0) name(g1, replace) nodraw
```

```
twoway (rarea nnt43s_lci nnt43s_uci temp, sort) (line nnt43s temptime, sort), legend(off) yline(0) name(g2, replace) nodraw
twoway (rarea nnt21a_lci nnt21a_uci temp, sort) (line nnt21a temptime, sort), legend(off) yline(0) name(g3, replace) nodraw
twoway (rarea nnt43a_lci nnt43a_uci temp, sort) (line nnt43a temptime, sort), legend(off) yline(0) name(g4, replace) nodraw
graph combine g1 g2, name(g5, replace) nodraw
graph combine g3 g4, name(g6, replace) nodraw
graph combine g5 g6, rows(2)

//Table 5 hausman specification test:
stset sht_time, failure(symHT==1)
streg Group ht_risk, distribution(gompertz) nolog nohr
estimates store gom1
stcox Group ht_risk, nolog efron nohr
estimates store cox1
stset, clear
stset stset any_time, failure(AnyHT==1)
streg Group ht_risk, distribution(gompertz) ancillary (ht_risk) nolog
estimates store gom2
stcox Group ht_risk, nolog efron nohr
estimates store cox2
hausman cox1 gom1, equ(1:1)
hausman cox1 cox2, equ(1:1)
hausman cox1 gom2, equ(1:1)
hausman gom1 cox2, equ(1:1)
hausman gom1 gom2, equ(1:1)
hausman cox2 gom2, equ(1:1)
drop _est*

//Table 6 for Cerebrolysin treatment effects
* HR
stset, clear
qui: stset sht_time, failure(symHT==1)
streg Group ht_risk, distribution(gompertz) nolog
qui: stset any_time, failure(AnyHT==1)
streg Group ht_risk, distribution(gompertz) ancillary (ht_risk) nolog
* Romano-Wolf p-values for multiple hypothesis testing:
stset, clear
qui: stset any_time, failure(AnyHT==1)
rwolf2 (streg Group ht_risk, distribution(gompertz) ancillary (ht_risk)), indepvars(Group) reps(1000) seed(50) nodots
stset, clear
qui: stset sht_time, failure(symHT==1)
rwolf2 (streg Group ht_risk, distribution(gompertz)), indepvars(Group) reps(1000) seed(100) nodots
* dRMST, symptomatic HT, HTI = 0
list rmst_diff21_lci rmst_diff21_uci rmst_diff21 temptime  if temptime==14
//calculating P-value from confidence intervals:
```

```
* go to online p-value calculator at:
https://www.graphpad.com/quickcalcs/pValue1/
* use option calculate p-value from z-score
* use computed z-score to obtain p-value
* di "se = " ([ul] - [ll])/(2*1.96)
* di "z = " [est] / [se]
di "se = " ([-.1200421724381] - [.6351652012232])/(2*1.96)
di "z = " [.2575615143926] / [-.19265494]
* dRMST, symptomatic HT, HTI > 0
list rmst_diff43_lci rmst_diff43_uci  rmst_diff43 temptime if
temptime==14
di "se = " ([3.087850400593] - [.5014517477602])/(2*1.96)
di "z = " [1.794651074177] / [-.65979557]
* dRMST, any HT, HTI = 0
list rmsta_diff21_lci rmsta_diff21_uci rmsta_diff21 temptime if
temptime==14
di "se = " ([-.0450624272614] - [.8059469145287])/(2*1.96)
di "z = " [.3804422436336] / [-.21709422]
* dRMST, any HT, HTI > 0
list rmsta_diff43_lci rmsta_diff43_uci rmsta_diff43 temptime if
temptime==14
di "se = " ([.1411963783634] - [3.855345274846])/(2*1.96)
di "z = " [1.998270826605] / [-.94748696]
* dSurvival, symptomatic HT, HTI = 0
list sdiff21_lci sdiff21_uci sdiff21 temptime if temptime==14
di "se = " ([-.0092652505596] - [.0491129269519])/(2*1.96)
di "z = " [.0199238381962] / [-.01489239]
* dSurvival, symptomatic HT, HTI > 0
list sdiff43_lci sdiff43_uci sdiff43 temptime if temptime==14
di "se = " ([.0385917568703] - [.2377442713731])/(2*1.96)
di "z = " [.1381680141217] / [ -.05080421]
* dSurvival, any HT, HTI = 0
list sadiff21_lci sadiff21_uci sadiff21 temptime if temptime==14
di "se = " ([-.0034622897619] - [.0760936099442])/(2*1.96)
di "z = " [.0363156600912] / [  -.02029487]
* dSurvival, any HT, HTI > 0
list sadiff43_lci sadiff43_uci sadiff43 temptime if temptime==14
di "se = " ([.0107783698932] - [.300609053985])/(2*1.96)
di "z = " [.1556937119391] / [  -.0739364]
* NNT, symptomatic HT, HTI = 0
list nnt21s_lci nnt21s_uci nnt21s temptime if temptime==14
di "se = " ([-23.34055379078] - [123.7228185085])/(2*1.96)
di "z = " [50.19113235884] / [ -37.516166]
* NNT, symptomatic HT, HTI > 0
list nnt43s_lci nnt43s_uci nnt43s temptime if temptime==14
di "se = " ([2.021526873114] - [12.45360337113])/(2*1.96)
di "z = " [7.237565122123] / [  -2.661244]
* NNT, any HT, HTI = 0
list nnt21a_lci nnt21a_uci nnt21a temptime if temptime==14
di "se = " ([-2.625279377214] - [57.69793935311])/(2*1.96)
di "z = " [27.53632998795] / [ -15.388576]
* NNT, any HT, HTI > 0
```

```
list nnt43a_lci nnt43a_uci nnt43a temptime if temptime==14
di "se = " ([.444642468063] - [12.40109147386])/(2*1.96)
di "z = " [6.422866970962] / [  -3.0501145]
//population attributable fraction:
stset, clear
qui: stset sht_time, failure(symHT==1)
*symptimatic Ht, all patients
qui: streg Group ht_risk, distribution(gompertz) nolog vce(robust)
punafcc, at(Group==1) eform vce(unconditional)
*symptomatic HT, patients with HTI>0
qui: streg Group if ht_risk==1, distribution(gompertz) nolog vce(robust)
punafcc, at(Group==1) eform vce(unconditional)
stset, clear
qui: stset any_time, failure(AnyHT==1)
*any HT, all patients
qui: streg Group ht_risk, distribution(gompertz) ancillary (ht_risk) nolog vce(robust)
punafcc, at(Group==1) eform vce(unconditional)
*any HT, patients with HTI>0
qui: streg Group if ht_risk==1, distribution(gompertz) nolog vce(robust)
punafcc, at(Group==1) eform vce(unconditional)

//Figure 10 hazard dynamics curves for symptomatic HT (see below, step 6):
*generating hazard acceleration curves and nonlinear hazard acceleration curves with 95% CI for sympt HT:
forvalues j = 1/4 {
dydx h`j' temptime, gen (dydx_h`j')
gen ac_h`j' = -(dydx_h`j')
gen nac_h`j' = -(dydx_h`j') + (h`j')^2
drop dydx_h`j'
dydx h`j'_lci temptime, gen (dydx_h`j'_lci)
gen ac_h`j'_lci = -(dydx_h`j'_lci)
gen nac_h`j'_lci = -(dydx_h`j'_lci) + (h`j'_lci)^2
drop dydx_h`j'_lci
dydx h`j'_uci temptime, gen (dydx_h`j'_uci)
gen ac_h`j'_uci = -(dydx_h`j'_uci)
gen nac_h`j'_uci = -(dydx_h`j'_uci) + (h`j'_uci)^2
drop dydx_h`j'_uci
}


*******************************************************************************
*****GENERATING 5% PEAK NLHA (95% CI LOWER BOUND) THRESHOLD AND ITS 95% CIs******
//STEP 0 — REQUIRED VARIABLES CHECK
*Before doing anything, make sure your dataset contains these variables:
    *   nac_h3 (mean NLHA)
```

```
    *   nac_h3_lci (95% lower CI NLHA)
    *   nac_h3_uci (95% upper CI NLHA)
    *   temptime (time from 1 to 14)
*Run:
describe nac_h3 nac_h3_lci nac_h3_uci temptime
*If all exist → continue

//STEP 1 — Compute SE of NLHA at each timepoint
*This is needed for the delta method:
gen double se_nlha = (nac_h3_uci - nac_h3_lci) / (2*1.96)

//STEP 2 — Identify the peak NLHA (lower bound) and the time it
occurs
*This is your base for the threshold:

* Find peak value of the 95%-CI lower-bound NLHA curve
quietly egen double peakval = max(nac_h3_lci)
scalar NLHA_peak_lb = peakval[1]

* Mark rows at the peak (in case of ties)
gen byte peak_flag = (nac_h3_lci == NLHA_peak_lb)

* Choose earliest time among tied peaks
quietly summarize temptime if peak_flag, meanonly
scalar tstar = r(min)

display "Peak NLHA lower bound = " NLHA_peak_lb
display "Peak occurs at time = " tstar

//STEP 3 — Extract the SE of NLHA at the peak time
*Use summarize rather than indexing:
quietly summarize se_nlha if temptime == tstar, meanonly
scalar SE_peak = r(mean)

display "SE of NLHA at the peak = " SE_peak

//STEP 4 — Delta method to get threshold estimate and 95% CI
*Your threshold formula:
*theta = 0.05 x NLHA_peak_lb
*Compute point estimate, SE, and CI:

* Proportion used for threshold (5%)
scalar p = 0.05

* Point estimate
scalar theta_est = p * NLHA_peak_lb

* Delta-method SE
scalar SE_theta = p * SE_peak

* 95% CI for the threshold
scalar theta_L = theta_est - 1.96 * SE_theta
```

```
scalar theta_U = theta_est + 1.96 * SE_theta

display "Threshold point estimate = " theta_est
display "Threshold 95% CI = [" theta_L ", " theta_U "]"

//STEP 5 — Create variables for graphing the horizontal threshold band
*These will be constant across all timepoints:
gen double threshold_mean = theta_est
gen double threshold_lci  = theta_L
gen double threshold_uci  = theta_U

//STEP 6 — Plot the NLHA curve + threshold CI band
*figure 10 combo
gen temptime7 = temptime if temptime<=7
twoway (line h3_lci ac_h3_lci nac_h3_lci  h3 ac_h3 nac_h3 temptime7, sort) (rarea threshold_lci threshold_uci temptime7, sort) (line threshold_mean temptime7, sort), ytitle("HT probability") xtitle("Analysis time, d") legend(order(4 "Hazard""function" 5 "Hazard""acceleration" 6 "NLHA")) name(g1, replace)
twoway (line nac_h1_lci nac_h2_lci nac_h3_lci nac_h4_lci nac_h3 nac_h4 temptime7, sort) (rarea threshold_mean threshold_uci temptime7, sort) (line threshold_mean temptime7, sort), ytitle("HT probability") xtitle("Analysis time, d") legend(order(1 "Control,""low HT risk" 2 "Cerebrolysin,""low HT risk" 5 "Control,""high HT risk" 6 "Cerebrolysin,""high HT risk")) name(g2, replace)
graph combine g1 g2

*****************************************************
//Table 8 therapeutic windows
forvalues j = 1/4 {
egen window_start`j' = min(cond(nac_h`j'_lci<=threshold_uci, temptime, .))
egen window_end`j' = min(cond(nac_h`j' <=threshold_mean, temptime, .))
summ window_start`j' window_end`j'
}

//Table 9 compouding effect
forvalues j = 1/4 {
egen window_endmax_quad`j' = min(cond(quad_h`j'_uci <= threshold_mean, temptime, .))
egen window_endmin_quad`j' = min(cond(quad_h`j'_uci <=threshold_uci, temptime, .))
summ window_endmin_quad`j' window_endmax_quad`j' quad_h`j' quad_h`j'_lci quad_h`j'_uci
}

//Sensitivity analysis of NLHA thresholds, TABLE 7:
summ nac_h3_lci
local peak = r(max)
```

```
local peak = r(max)
local thr2 = `peak'*0.02
local thr5 = `peak'*0.05
local thr10 = `peak'*0.10
local thr13 = `peak'*0.13
egen inception_2 = min(cond(nac_h3_lci<=`thr2', temptime, .))
egen inception_5 = min(cond(nac_h3_lci<=`thr5', temptime, .))
egen inception_10 = min(cond(nac_h3_lci<=`thr10', temptime, .))
egen inception_13 = min(cond(nac_h3_lci<=`thr13', temptime, .))
egen inception_2m = min(cond(nac_h3 <=`thr2', temptime, .))
egen inception_5m = min(cond(nac_h3 <=`thr5', temptime, .))
egen inception_10m = min(cond(nac_h3 <=`thr10', temptime, .))
egen inception_13m = min(cond(nac_h3 <=`thr13', temptime, .))
summ inception_2 inception_5 inception_10 inception_13
summ inception_2m inception_5m inception_10m inception_13m

//FIGURE 11 combo:
*generating quadratic hazard function and its log-transformation:
forvalues j=1/4 {
gen quad_h`j' = h`j'^2
gen quad_h`j'_uci = h`j'_uci^2
gen quad_h`j'_lci = h`j'_lci^2
gen log_quad_h`j' = log(quad_h`j')
gen log_quad_h`j'_uci = log(quad_h`j'_uci)
gen log_quad_h`j'_lci = log(quad_h`j'_lci)
gen log_nac_h`j' = log(nac_h`j')
gen log_nac_h`j'_lci = log(nac_h`j'_lci)
gen log_nac_h`j'_uci = log(nac_h`j'_uci)
}
*generating thresholds
gen log_threshold_mean = log(threshold_mean)
gen log_threshold_lci = log(threshold_lci)
gen log_threshold_uci = log(threshold_uci)
*generating graph 11:
twoway (rarea log_quad_h1_lci log_quad_h1_uci temptime, sort) ( rarea log_quad_h3_lci log_quad_h3_uci temptime, sort) (line log_quad_h1 log_quad_h3 log_nac_h3_lci log_nac_h3 temptime, sort) ( rarea log_threshold_mean log_threshold_uci temptime, sort) (line log_threshold_mean temptime, sort),legend(order(3 "Compounding effect, low HT risk" 4 "Compounding effect, high HT risk" 6 "NLHA, high HT risk")) name(g1)
twoway (rarea log_quad_h2_lci log_quad_h2_uci temptime, sort) ( rarea log_quad_h4_lci log_quad_h4_uci temptime, sort) (line log_quad_h2 log_quad_h4 log_nac_h4_lci log_nac_h4 temptime, sort) ( rarea log_threshold_mean log_threshold_uci temptime, sort) (line log_threshold_mean temptime, sort),legend(order(3 "Compounding effect, low HT risk" 4 "Compounding effect, high HT risk" 6 "NLHA, high HT risk")) name(g2, replace)
grc1leg g1 g2, legendfrom(g1)
```
